# Brain Connectivity Studies on Structure-Function Relationships: A Short Survey with an Emphasis on Machine Learning

**DOI:** 10.1155/2021/5573740

**Published:** 2021-05-27

**Authors:** Simon Wein, Gustavo Deco, Ana Maria Tomé, Markus Goldhacker, Wilhelm M. Malloni, Mark W. Greenlee, Elmar W. Lang

**Affiliations:** ^1^CIML, Biophysics, University of Regensburg, Regensburg 93040, Germany; ^2^Experimental Psychology, University of Regensburg, Regensburg 93040, Germany; ^3^Center for Brain and Cognition, Department of Technology and Information, University Pompeu Fabra, Carrer Tanger, 122-140, Barcelona 08018, Spain; ^4^Institució Catalana de la Recerca i Estudis Avançats, University Barcelona, Passeig Lluís Companys 23, Barcelona 08010, Spain; ^5^IEETA/DETI, University de Aveiro, Aveiro 3810-193, Portugal

## Abstract

This short survey reviews the recent literature on the relationship between the brain structure and its functional dynamics. Imaging techniques such as diffusion tensor imaging (DTI) make it possible to reconstruct axonal fiber tracks and describe the structural connectivity (SC) between brain regions. By measuring fluctuations in neuronal activity, functional magnetic resonance imaging (fMRI) provides insights into the dynamics within this structural network. One key for a better understanding of brain mechanisms is to investigate how these fast dynamics emerge on a relatively stable structural backbone. So far, computational simulations and methods from graph theory have been mainly used for modeling this relationship. Machine learning techniques have already been established in neuroimaging for identifying functionally independent brain networks and classifying pathological brain states. This survey focuses on methods from machine learning, which contribute to our understanding of functional interactions between brain regions and their relation to the underlying anatomical substrate.

## 1. Motivation

**Figure 1:**
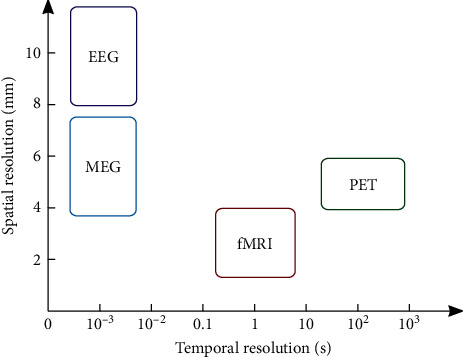
The temporal and spatial resolutions on which different functional neuroimaging techniques can operate. While fMRI allows to study neural processes at higher spatial resolution, EEG and MEG can better resolve neural activity dynamics in the temporal domain.

Similar to molecular biology, neuroscience also faces the problem to bridge the gap between experimental techniques, which study the anatomical substrate of neural information processing and techniques, used to determine functional interactions between specified brain regions. In molecular biology, it is known that such a relationship is only loosely linked such that, on the one hand, different 3D protein structures may have similar functions, while, on the other hand, similar 3D structures may exhibit rather different functions in the metabolism [1]. Still, as even single amino acid replacements may change the function of the protein completely, structure and function must be related to a certain extent. Similarly, in neuroscience, evidence has been collected over the last two decades suggesting that the anatomical structure of the neuronal network determines important constraints to the functional organization of neuronal activities and concomitant information processing [2–4]. Hence, they must be somehow interrelated. Moreover, though space interactions (such as, for example, electromagnetic fields or magnetic dipole-dipole interactions in physics) are not (yet) of relevance in neuroscience, information processing is confined to the anatomical substrate and thus depends on its structural connectome. Large-scale activity distribution is mediated via propagating action potentials; therefore, the spatial organization of neuron assemblies and their dendritic and axonal connections forms the underlying physical substrate for information processing. Experimental evidence for neural network topologies mainly comes from noninvasive neuroimaging techniques and neuroanatomical methods, while their functional variants consider the related inherent dynamics. Formerly, only the static aspect of this organization has been studied, while recent evidence demonstrated the importance to also consider the highly dynamic nature of functional activity patterns. Functional neuroimaging techniques such as functional magnetic resonance imaging (fMRI), electroencephalography (EEG), magnetoencephalography (MEG), or positron emission tomography (PET) operate on several distinct spatiotemporal scales, and an overview of the respective spatial and temporal resolutions is provided in Table 1. The relation of the scales which can be covered by these different neuroimaging techniques is further illustrated in Figure 1. Moreover, understanding processing of information relies on the applied physical modeling and simulation, statistical analysis, signal processing, and, more recently, also machine learning techniques. This noncomprehensive survey explores the recent literature on these issues and advocates for the idea of pursuing both modeling and data-driven analysis in combination.

A better understanding in the relationship between the brain structure and function can provide insights into the integrated nature of the brain. Firstly, such an approach can contribute to our understanding of how information is first segregated and then integrated across different brain regions, while it could explain how complex neural activity patterns emerge, even in a resting brain [5, 6]. Moreover, as brain connectivity can be revealed by various statistical measures and different imaging modalities, all these follow their individual concepts displayed on different spatial and temporal scales. Understanding the interplay between the structure and function can help to interpret what can actually be seen in data obtained by different brain imaging modalities. Such an analysis can tell us how to relate these different modalities to each other [3, 4] and further how to integrate them in a meaningful manner [7, 8]. Finally, such integrated models can be of clinical relevance, for example, by explaining how structural lesions affect the brain not only locally but also via functional connections across the whole brain [9, 10].

## 2. Complex Brain Networks

The brain is organized into spatially distributed but functionally connected regions of dynamically correlated neuronal activity. These dynamically changing network structures can be characterized by three different but related forms of connectivity [11]:

- *Structural connectivity* (SC) via excitatory and inhibitory synaptic contacts gives rise to the so-called connectome [12, 13]. Modern neuroimaging technologies, especially diffusion tensor imaging (DTI), provide the basis for the construction of structural graphs representing the spatial layout of white matter fiber tracks that serve to link cortical and subcortical structures. These graphs are characterized by densely connected nodes forming network hubs and fiber tracks (white matter) which connect spatially distant neuronal pools. The anatomy of this neuronal network exhibits substantial plasticity on long time scales, usually due to its natural development, aging, or disease [14, 15], though it is quite stable for short time scales. Therefore, it is considered as static in most of the experiments [16].
- *Functional connectivity* (FC) expresses temporal correlations between neuronal activity patterns occurring simultaneously in spatially segregated areas of the brain. Such temporal activity patterns can be encoded in functional graphs and quantified through statistical concepts. They fluctuate on multiple time scales ranging from milliseconds to seconds. FC patterns are robustly expressed by resting-state networks (RSNs), where they emerge from spontaneous neuronal activity. They exhibit complex spatiotemporal dynamics, which have been described within the realm of state-space models by frequent transitions between discrete FC configurations. Much effort has been spent to characterize the latter. However, understanding the mechanisms that drive these fluctuations is more elusive and has recently been the subject of intense modeling efforts [17]. Although these fluctuations have long been considered stationary, recently, it became obvious that the consideration of their nonstationary nature is essential for a thorough understanding of information processing in the brain [2]. Furthermore, it remains elusive to what extent such functional graphs map onto structural graphs, i.e., how the network dynamics are constrained by the underlying anatomy.
- *Effective connectivity* (EC) or *directed connectivity* describes causal interactions between activated brain areas [18]. As correlation does not imply causation, these concepts were established to deal with the directional influences of segregated neuron assemblies. If inferred from time series analysis, Granger causality [19] does not need any information about the structural organization of the neuronal network. Additionally, dynamic causal modeling (DCM) provides a deterministic model of neural dynamics, describing causal mechanisms within brain networks [20]. Recent investigations showed that FC patterns can be modeled successfully if global dynamic brain models are constrained by EC rather than by SC [21].

**Figure 2:**
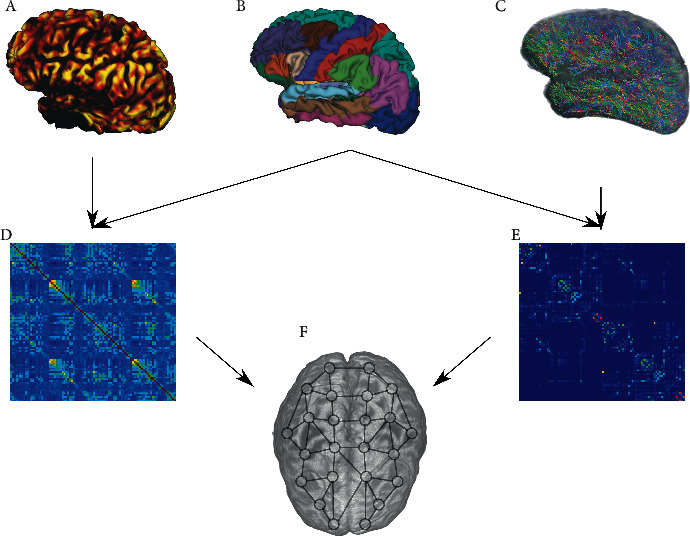
In fMRI, spatial-temporal activity maps of the human brain can be observed (A). On the contrary, DTI can be used to model white matter connections between different spatial brain regions (C). Next, a set of brain regions can be defined to act as nodes in a brain network (B). By quantifying the temporal coherence of activity fluctuations in a group of brain regions (B), the strength of functional connectivity (FC) is derived and can be arranged in a FC matrix (D). Analogous thereto, structural connectivity (SC) can be described by measuring the anatomical connection strength between those regions (B) and can be combined to a SC matrix (E). These two connection profiles can complement each other to give us a more comprehensive picture of brain connectivity (F).

Modern imaging techniques map out structural and functional connectivities with remarkable spatial and temporal resolutions. A summary of how structural and functional connectivity can be conceptually derived from MRI is provided in Figure 2. While static structural connectivity is straightforward, its functional counterpart is more subtle. Current views consider two distant brain regions as functionally connected if their activity fluctuates synchronously and coherently, implying an instantaneous and persistent constant phase relation between their temporal activities. Given a pair of distinct brain areas (regions of interest, neuron pools, and network nodes), their SC is often derived from diffusion tensor imaging (DTI), high angular resolution diffusion imaging (HARDI), and diffusion spectrum imaging (DSI). Structural connection strength is typically quantified experimentally by the number of fibers, fractional anisotropy, etc., and these values are converted into edge weights in graphical models [22]. On the contrary, FC is derived from fMRI, EEG, or MEG and quantified by static measures such as instantaneous cross-correlations or partial correlations, while dynamic measures consider coherence and Granger causality [23].

The analysis of these types of network connectivity leads to the notion of complex brain networks [24]. Intense efforts have been exerted over the last decade to unravel the mechanisms and principles of neuronal information processing and to bridge the gap between the different types of connectivity analysis. The timely review by Friston [25] detailed biophysical concepts used to model such connectivities. Again, the push came from neuroimaging techniques such as DTI [26], which allow us to track fibers, though at relatively low spatial resolution only. In the postmortem human brain, 3D polarized light imaging (3D-PLI) [27] allows us to trace the 3-dimensional course of fibers with a spatial resolution even of the micrometer range.

While these connectivity concepts were being developed, doubts began to be raised concerning the usefulness of the concept of functional or effective connectivity as long as its relation to structural connectivity is not sufficiently understood [28]. The concerns mainly focused on different spatial and temporal scales, with which functional/effective versus structural connectivity was determined using different neuroimaging modalities.

## 3. Graphical Models of Brain Networks

Graphical models represent physical variables as a set of possibly connected nodes, also called vertices, and related edges, which signal marginal or conditional interactions between the connected nodes. Such models are commonly used to describe complex interrelationships between the set of variables. The central idea is that each variable, for example, the neuronal activity of a localized neuron pool, is represented by a node in a graph. Such nodes may be joined by edges of variable strength. Hence, the topology of complex brain networks can be characterized by static (GM) or dynamic graphical models (DGM), either on a structural or on a functional basis [29–34]. They represent a versatile mathematical framework for a generic study of pairwise relations between interacting brain regions. Small-world networks (SWNs) [35] provide an adequate description of the static topology of brain networks. Such SWNs exhibit the two principles of segregation and integration of information processing in the brain.

Given that the functional organization of the brain changes along various intrinsic time scales, graphical models also need to be dynamic descriptors of these spatiotemporally fluctuating neuronal populations. As DGMs are applicable across various spatiotemporal scales, they most adequately represent the temporal complexity of the activity of interacting neuronal populations. Recent studies of DGMs elaborate on inferring FC from SC and vice versa. They describe metrics suitable for quantifying the SC-FC interrelationship by elucidating the eigenspectrum of structural Laplacian (Given a graph, its Laplacian measures the difference of the diagonal matrix of its vertex degrees and its adjacency matrix (see Appendix A). A few common eigenmodes are indeed sufficient to reconstruct FC matrices. Closely related techniques employ independent component analysis (ICA) of FC time courses [36]. Many of these studies also emphasize the decisive role of indirect structural connections, for example, based on spectral mapping methods. Most of these studies are based on linear DGMs because of their computational stability and ease to infer reverse correlations. Studies aimed to predict SC from FC are considered as well because DTI data sometimes fail to model certain anatomical connections [37]. Other interesting issues that have been addressed concern the subject specificity of SC-FC relationships and their dependence on the employed imaging modalities.

DGMs can promote our understanding of emotional and cognitive states, task switching, adaptation and development, or aging and disease progression. Graph theory thus provides a comprehensive description of topological and dynamical properties of complex brain networks. Most studies seem to corroborate that network topology, to a large extent, determines time-averaged functional connectivity in large-scale brain networks. However, not many tools yet exist to describe dynamic graphs, and only very few consider the relationship between static and dynamic graphical models.

Tools for such analysis are made available for researchers, such as GraphVar (https://www.nitrc.org/projects/graphvar/) [38, 39], a GUI-based toolbox for comprehensive graph theoretical analyses of brain connectivity, as well as the construction, validation, and exploration of machine learning models. Dynamic graphical models have also been developed and are available in R (https://github.com/schw4b/DGM) [34]. Such DGMs can deal with spatiotemporal activity patterns, allow for loops in networks, and can describe directions of instantaneous couplings between nodes. Furthermore, temporal lags of the hemodynamic response between coupled nodes have been shown to influence quantitative directionality estimates but are known for avoiding false positive estimates [34].

Very recently, Meunier et al. [40] presented NeuroPycon (https://github.com/neuropycon), an open-source toolbox for advanced connectivity and graph theoretical analysis of MEG, EEG, and MRI data. Often, one problem is the reproducibility of processing pipelines in neuroimaging studies. To tackle this problem, the NeuroPycon toolbox wraps commonly used neuroimaging software for processing and graph analysis into a common Python environment and provides shareable parameter files. Currently, NeuroPycon offers two packages named ephypype and graphpype. While the former focuses on EEG and MEG data analysis, the latter is designed to study functional connectivity employing graph theoretic metrics. Accordingly, this open-source software package can help to facilitate sharing and reproducing scientific results for the neuroscience community.

### 3.1. Graph Topology of Brain Networks

With respect to complex network architectures, an especially attractive network topology is characterized by the so-called small-world organization of complex systems [35, 41]. Such small-world networks (SWN) are characterized by densely connected nodes of information processing which are distant in the anatomical space and only sparsely connected via long-range connections between different functionally interacting brain regions. Their main characteristic is reflected in high clustering (similar to that found for a regular lattice) and low path length (similar to a random network). Such topology allows efficient information processing at different spatial and temporal scales with a very low energy cost [42]. Note that such networks are sometimes liberally classified as SWNs, implying their unique properties, but they are lacking essential characteristics of SWNs [43]. Specifically, clustering in networks needs to be compared to clustering on the lattice and not random networks. Telesford et al. [43] proposed a proper metric for such comparison. If brain networks show a small-world network topology, it is mirrored by two principles of information processing: functional segregation on small, quasi-mesoscopic spatiotemporal scales but functional integration on larger, macroscopic spatial and temporal scales.

Recently, Sizemore and Bassett [31] reviewed existing methods and employed a publicly available MATLAB toolbox (https://github.com/asizemore/Dynamic-Graph-Metrics) to visualize and characterize such dynamic graphs with proper metrics. In [44], Sizemore et al. already showed that the algebraic topology is well suited to characterize mesoscale structures of brain connectivity formed by cliques (a set of adjacent vertices) in an otherwise sparsely connected network. Aside from cliques, topological network cavities of varying sizes were observed to link regions of early and late evolutionary origin in long loops, presumably playing an important role in controlling the brain function. Differences in the topological organizations of functional and structural graphs were the focus in the study by Lim et al. [45], who adopted a multilayer framework with SC and FC. Their analysis showed that SC tends to be organized such that brain regions are mainly connected to other brain regions with similar node strengths, while FC shows smaller values of assortativity [46], which can be associated with robustness of brain functions in the context of network theory [45].

### 3.2. Graph Theoretical Aspects of SC-FC Relations

Independence tests have shown strong correlations between structural and functional connectivities [47]. As shown by Hermundstad et al. [3], the length, number, and spatial location of anatomical connections (SCs) can be inferred from the strength of the resting state and task-based functional correlations (FCs) between brain regions. With resting-state networks (RSNs), FC is constrained by the large-scale SC of the brain in terms of strength, persistency, and spatial statistics. Often, functionally connected brain areas do not show any direct anatomical connections, pointing to the importance of indirect connections as well, for example, via the thalamus. This discrepancy between structural and functional connectivity also motivates us to combine these measures in order to overcome shortcomings of individual measures and to get a more comprehensive picture of brain connectivity [7, 8]. In an early study, Honey et al. [4] concluded from computational modeling that the inference of structural connectivity from functional connectivity deems impractical. Still, several recent graph theoretical studies elaborate on inferring FC from SC and vice versa. They provide different metrics for quantifying the SC-FC interrelationship and discuss the decisive role of indirect connections.

Abdelnour et al. [48] added a new twist to inferring FC from SC by considering linear computational GMs rather than the commonly employed nonlinear simulations. They captured the long-range second-order correlation structure of RSNs, which governs the relationship between its anatomic and functional connectivities. The model applied random walks on a graph (see Appendix A) to structural networks, as deduced by DTI, and predicted the FC structure obtained from the fMRI data of the same subjects. Because of its linearity, the model also allows inverse predictions of SC from FC. The study thus corroborates the linearity of ensemble-averaged brain signals and suggests a percolation model (Percolation theory describes the behavior of connected clusters in a random graph.) [49], where purely mechanistic processes of the structural backbone confine the emergence of large-scale FCs. Yet, another linear model of correlations across long-duration BOLD fMRI time series has been devised by Luisa Saggio et al. [50] to measure FC for the comparison of real and simulated data. The analytically solvable model considers the diffusion of physiological noise along anatomical connections and provides FC patterns from related SC patterns. The model allows for the investigation of nonstationary temporal dynamics in RSNs and, because of its linearity, can be inverted easily to deduce SC from known FC. Later, Abdelnour et al. [51] further investigated the interplay of SC versus FC on graphs by elucidating the eigenspectrum of structural Laplacian (see Appendix A). The authors showed that both SC and FC share common eigenvectors, their eigenvalues are exponentially related, and a small number of eigenmodes are sufficient to reconstruct FC matrices. The method is intimately related to a data-driven independent component analysis (ICA) of FC time courses and outperforms time-consuming generative simulations of dynamic brain network models. The importance of FC time courses was also emphasized in the study of Sarkar et al. [52]. As anatomical connections are often missing from the DTI data, the authors considered the inverse problem of inferring SC from FC and formulated it as a convex optimization problem regularized with sparsity constraints based on physiological observations. The study could not only reproduce quantitative measures of SC on a fine-grained cortical dataset, consisting of 998 nodes, but also robustly predict long-range transhemispheric couplings, which are not resolved by DTI.

Concerning computational GMs, Huang and Ding [56] also considered the question of proper quantification of SC-FC interrelationships. They showed that conditional Granger causality (cGC) was significantly correlated across subjects with edge weights [22] in RSNs, but not with mean fractional anisotropy. The authors concluded that edge weight represents the proper SC measure, while cGC adequately measures FC. Following the question of proper quantification of FC, Meier et al. [57] compared the structure-function relationship, when using different imaging modalities to assess FC, measured not only with fMRI but also with MEG. The study mainly considered local connectivities between immediate neighbors, with some connections in homologous regions of the opposite hemisphere. The results of their SC-FC mapping indicated that, although sharing many properties, the SC-FC relationship also seems to be imaging modality dependent. Following this hypothesis, the idea of employing imaging modalities with higher temporal resolution for observing brain functions, also with state-of-the-art tractography methods for reconstructing the brain structure, is an interesting direction for future research on this field [16].

**Figure 3:**
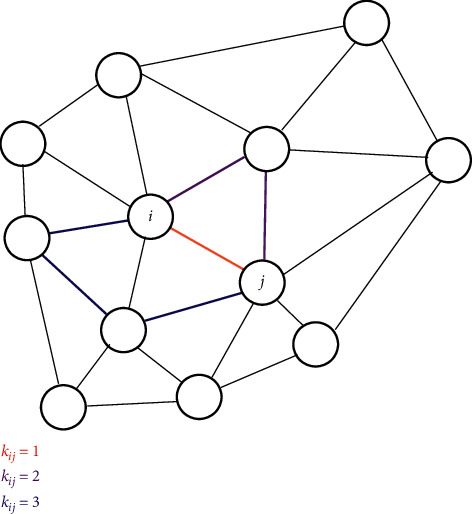
In the most simple case, structural connections between two brain areas i and j could be direct (order *k*_*ij*_=1), but also, higher-order connections (*k*_*ij*_=2,3,…) between two regions play a significant role for the propagation of neural signals [53–55].

Topological aspects of the SC-FC relationship were further discussed by Liang and Wang [55] at the level of connectivity matrices. They developed a similarity measure to assess the quality of various network models based on persistent homology and regularized the solution to cope with large matrices. The measure could distinguish between direct and indirect SCs for predicting FC. The results corroborated a nonlinear structure-function relationship and suggested that the FC in RSNs is characterized not only by direct structural connections but also by sparse indirect connections.  Figure 3 illustrates such higher-order connections between two nodes in a network. Also, Becker et al. [53] investigated how brain activity propagates along indirect structural walks by using a spectral mapping method (Spectral graph theory is the study of properties of the Laplacian matrix or adjacency matrix associated with a graph.) [58], which systematically reveals how the length of indirect structural walks in a network influences the FC between two nodes. Results of their mapping suggested that walks on the structural graph up to a length of three contribute most to the functional correlation structure. Such indirect connections were also the focus of the work of Røge et al. [59]. Simulations on high-resolution whole-brain networks show that functional connectivity is not well predicted by direct structural connections alone. However, predictions improved considerably if indirect structural connections were integrated. The authors also showed that shortest structural pathways connecting distant brain regions represent good predictors of FC in sparse networks. This focus on the shortest structural paths between connected brain regions was also taken up by Chen and Wang [60], who designed an efficient propagation network built with only the shortest paths between brain regions. Concerning subject specificity of SC-FC relations, Zimmermann et al. [61] furthermore showed that because of the marginal variability between subjects in SCs compared to a rather pronounced variability in FCs, little subject specificity results, indicating that FC is only weakly linked to SC across subjects.

Bettinardi et al. [54] considered the postulate that coactivated brain areas should have similar input patterns and elaborated on this idea to explain how anatomical connectivity can determine the spontaneous correlation structure of brain activity. They explored the idea that information, once generated, spreads rather isotropically along all possible pathways while decaying in strength with increasing distance from its origin. The authors analytically quantified the similarity of whole-network stimulus patterns based solely on the underlying network topology, thus generalizing the well-known matching index [62]. Finally, the authors could corroborate that the network topology, to a large extent, determines time-averaged functional connectivity in large-scale brain networks.

In an effort to explain brain dynamics, Gilson et al. [63] considered a multivariate Ornstein–Uhlenbeck model (An Ornstein–Uhlenbeck process is a stochastic stationary Gauss–Markov process, which solves the Langevin equation.) [64] on a DGM to estimate statistics characterizing effective connectivities (ECs) in RSNs. Linear response theory (LRT) (Let *x*(*t*) be a stimulus and *y*(*t*) a related system response; then, both are related by *y*(*t*) ≈ ∫_−*∞*_^*t*^*χ*(*t* − *t*′)*x*(*t*′)d*t*′, where the susceptibility *χ*(*t* − *t*′) represents the linear response function. The latter is related to Green's function in case of a Dirac delta impulse stimulus.) was then employed to estimate network-related Green's function, which specifies the dynamic coupling between network nodes. The model provided graph-like descriptors (community and flow) to describe the role of either nodes or edges to propagate activity within the network. The graphical model thus stresses temporal aspects, which merge segregated functional communities to integrate into a global network activity. The approach is not limited to resting-state dynamics but can also deal with task-evoked activity.

The interrelation between connectivity, as derived from different imaging modalities, was studied in a systematic approach by Garcés et al. [65]. They investigated similarities between SC derived from DTI, FC observed in fMRI, and FC measured in MEG on different spatial scales: global network, node, and hub level. They verified the strong relation between SC and FC observed in MRI, but also found strong similarities between SC and FC in MEG at theta, alpha, beta, and gamma bands. In their analysis, they could find the highest node similarity across modalities in regions of the default mode network and the primary motor cortex. The relation between structural and functional graphs was further exploited by Glomb et al. [66] to overcome problems in FC-EEG analysis. In whole-cortex EEG studies, volume conduction can induce spurious FC patterns, which are hard to disentangle from genuine FC. Glomb et al. [66] proposed a technique to smooth EEG signals in the space defined by white matter connections, in order to strengthen the FC between structurally connected regions, which could improve the resemblance of FC observed with EEG and FC measured with fMRI.

## 4. Computational Connectomics

Functional neuroimaging techniques initiated connectome-based computational modeling of brain networks, called computational connectomics (CC). The latter reproduces experimental findings related to such large-scale activity distributions. Furthermore, such modeling also encompasses spatiotemporal multiscale concepts of information processing in such complex networks. To ease such modeling endeavors, simulation platforms such as the Brain Dynamics Toolbox (https://bdtoolbox.org/) [24, 67] or DynamicBC (http://www.restfmri.net/forum/DynamicBC) [68] have been developed which support the major classes of differential equations of interest in computational neuroscience and/or implement both dynamic functional and effective connectivities for tracking brain dynamics from functional MRI. On a more phenomenological level, The Virtual Brain (https://www.thevirtualbrain.org/tvb/zwei) neuroinformatics platform [69, 70] provides a brain simulator as an integrated framework which encompasses several neuronal models and their dynamics. It offers multiscale model-based simulations and allows inference of neurophysiological processes underlying functional neuroimaging datasets. Hence, such modeling frameworks generate features, which allow for an understanding of underlying mechanisms beyond computational reproduction.

One typical interpretation derived from computational modeling is that the brain at rest resembles a dynamically metastable (A metastable state of a dynamical system is stable against small perturbations but not against large perturbations. It corresponds to a minimum of the free-energy landscape other than the global minimum. In neuroscience, it is sometimes more loosely used to denote a transient state of the system that persists for a finite lifetime only.) system with frequent switches between several metastable states, potentially driven by multiplicative noise [2]. By constraining computational models by the anatomical connections derived from DTI, they can serve as a link between the brain structure and correlation patterns, empirically observed in functional MRI. Usually, in such a framework, the strength of white matter connections characterizes the coupling strength of nodes in such large-scale computational models. Furthermore, with such simulations, it can be studied how different topological properties of the anatomical substrate contribute to the systems' dynamics, explaining how functional connectivity patterns depend on the underlying structural backbone [71, 72].

Recently, machine learning methods were also applied to extract characteristic features of the underlying networks from functional neuroimaging. These features opened the field for deducing functional connectivity from structural connectivity and vice versa in a purely data-driven approach [47, 73–76].

### 4.1. The Resting State as a Dynamically Metastable System

Though neuronal activity fluctuates continuously even without being driven by external stimuli, a thorough understanding of the spatiotemporal dynamics of complex brain networks is yet to be achieved. With the seminal paper of Raichle et al. [77], the notion of a default mode network (DMN) of a resting brain was born. This concept triggered a wealth of studies related to the resting state of the brain [2, 78, 79], whereby the resting brain is in general understood as the state in which the brain does not receive any explicit input. Such computational connectomics studies revealed that the resting human brain represents a quasi-metastable dynamical system [2] with frequent fluctuations around a dynamic equilibrium network state, which occasionally resembles the default mode network (DMN) of the resting state [77, 80]. Note that this equilibrium network state is not defined as a global minimum of an energy landscape like that used to model protein folding. Rather, it is understood as a steady-state balancing deployment of fast and slow systems which process neuronal activations. Such brain network modeling has the potential to reveal nontrivial network mechanisms and goes beyond canonical correlation analysis of functional neuroimaging. Indeed, the main driving force behind these computational brain network modeling efforts results from the observation that the spiking of single neurons is understood in biophysical detail, but how large-scale, whole-brain spatiotemporal activity dynamics emerge from spontaneously spiking neuron assemblies is still a matter of much debate, and their underlying mechanisms are only partly understood [78]. This is especially intriguing as even a resting brain without external stimuli shows highly structured spatiotemporal activity patterns far from being random.

### 4.2. The Statistical Mechanics Perspective of Brain Dynamics

Models adapted from statistical physics provide one possibility to describe brain dynamics empirically observed in different neuroimaging modalities such as fMRI. Concerning the resting state, in an early work, Fraiman et al. [81] focused on the question whether such a state can be comparable to any known dynamical state. For that purpose, correlation networks deduced from human brain fMRI investigations were contrasted with correlation networks extracted from numerical simulations of an Ising model (An Ising model describes an interacting lattice spin system with two degrees of freedom for every spin variable. Each spin interacts with its immediate neighbors and an external field. In 2D, an Ising model exhibits a phase transition from an unordered to an ordered phase. It represents one of the few exactly solvable models in statistical physics.) [82] in 2D, at different temperatures. Near the critical temperature *T*_*c*_, strikingly similar statistical properties rendered the two networks indistinguishable from each other. These results were considered to support the conjecture that the dynamics of the functioning brain is near a critical point.

**Figure 4:**
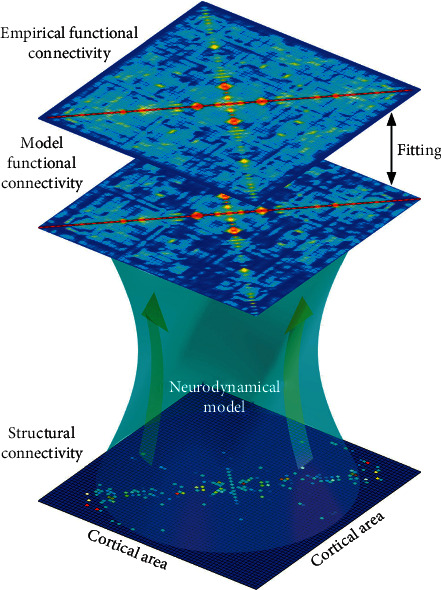
Structural connectivity (SC), like that derived from DTI, can be included to describe the couplings of nodes (anatomical areas) in a neurodynamical model, constraining the neural dynamics. The emerging functional connectivity (FC) patterns of the model can then be compared to the empirical FC obtained by fMRI (adapted from Deco et al. [84]).

Fortunately, all modeling efforts profit from having available constraints from functional, effective, and structural connectivity measures as provided by empirical neuroimaging data [83]. An early attempt to include such constraints was undertaken by Deco et al. [84], who derived a mean-field model (In the limit of a large number of interacting entities in a network, a Markov chain model of network dynamics is often replaced by a mean-field model, which assumes that each unit homogeneously interacts with the network in an average way only. An especially illusive example is mean-field approximations of the Ising model [85].) [86] (MFM) of the stationary dynamics of a conductance-based synaptic large-scale network of a spiking neuron population. The connectivity in this network was constrained by DTI data from human subjects, such as that illustrated in Figure 4. The temporal evolution of the neuronal ensemble was approximated by the longest time scale of the dynamic mean-field model. The latter has been further simplified into a set of equations of motion (An equation of motion describes the dynamic behavior of a physical system in terms of generalized coordinates as a function of time.) for statistical moments. These differential equations provided an analytical link between anatomical structure, stationary neural network dynamics, and FC. In a subsequent seminal paper of Deco at al. [2], nonstationary network dynamics have been considered as well. There, the resting brain has been modeled by a network of noisy Stuart–Landau oscillators (Stuart–Landau oscillators represent coupled limit-cycle oscillator, which exhibit collective behavior such as synchronization.), each operating near a critical Hopf bifurcation (An Andronov-Hopf bifurcation consists in the birth of a limit cycle out of an equilibrium point of a dynamic system such as coupled oscillators.) point in the phase space [87]. Each oscillator is running at its intrinsic frequency as given by the mean peak frequency of the narrowband BOLD signals of each brain region which the oscillator represents. Note that this ensemble of locally coupled oscillators is different from ensembles of nonlocally coupled, identical oscillators which show chimera states (Chimera states represent a unique collective behavior of a dynamic system, where coherent and incoherent states coexist.) which have attracted much attention recently [88–90]. Rather, in a Kuramoto-type (A Kuramoto model consists of a set of phase oscillators, which rotate at disordered intrinsic frequencies and with nonlinear couplings. Their time-dependent amplitudes are neglected.) [91, 92] approach, each oscillator is characterized through its phase variable only, while the time-dependent amplitude of the analytical signal is neglected. The resulting global network dynamics reveals spatial correlation patterns constrained by the underlying anatomical structure. By investigating cortical heterogeneity across the entire brain, the authors explained how fluctuations around a dynamic equilibrium state of a core brain, represented by eight identified brain areas, could drive functional network state transitions [2, 93, 94]. Hence, in the work of Naskar et al. [95], the resting brain is considered to represent a dynamically metastable system with frequent switches between several metastable states driven by multiplicative noise. Metastability can be quantified in such coupled oscillator systems by the standard deviation of one of the two time-dependent Kuramoto order parameters, which measures the phase coherence of a set of oscillators in the weak coupling limit [2]. In this respect, the brain can be considered as operating at maximal metastability suggesting some kind of spinodal-like (The spinodal curve in a phase diagram connects states where the second derivative of the Gibbs free energy is zero. At these points, even the faintest perturbation induces a phase transition to the related equilibrium state.) instability [96] in the phase space, where a transition towards a related stable, possibly task-related network state occurs as a consequence of a small external disturbance. It is interesting to see recently that research into coupled Stuart–Landau oscillator networks has also focused on the amplitude dynamics of the analytic signal [89, 90, 97]. It has been shown that an explosive death of oscillations can be observed which might be related to the suppression of neuronal activity in RSNs evoked by stimulus-driven information processing [98].

The idea of viewing human brain dynamics from a statistical mechanics perspective was also proposed by Ashourvan et al. [99]. However, rather than studying the evolution of regional activity between local attractors (In a dynamical system, an attractor represents a set of numerical values towards which the system evolves from a wide range of initial conditions. An attractor can be a point, a finite set of points, a curve, a manifold, or even a complicated set with a fractal structure known as a strange attractor.) representing mental states, time-varying states composed of locally coherent activity (functional modules) were put forward. A maximum entropy model was adapted to pairwise functional relationships between ROIs, based on an information-theoretic energy landscape model, whose local minima represent attractor states with specific patterns of the modular structure. Clustering such attractors revealed three types of functional communities. Transitions between community states were simulated using random walk processes. Thus, the brain is understood as a dynamical system with transitions between basins of attraction characterized through coherent activity in localized brain regions.

### 4.3. Spatiotemporal Brain Dynamics and Statistical State-Space Models

Although large-scale activity distributions in RSNs have long been considered stationary, recent investigations provided ample evidence of their nonstationary nature. Consequently, it is insufficient to only consider the grand average functional connectivity (FC); rather, its nonstationary dynamical nature has to be considered as well [2]. Several recent studies showed how functional connectivity states can emerge from a single stationary structural connectivity net through fluctuations around this metastable state. The latter can be described with mean-field models, while the fluctuations can be generated with an ensemble of coupled Stuart–Landau oscillators with simple attractor dynamics and reproduce the spatiotemporal connectivity dynamics if constrained by EC rather than SC as deduced from DTI measurements.

While the functional connectivity can be estimated via a linear Pearson correlation (Pearson's correlation coefficient is a measure of the linear bivariate correlation between two variables *x* and *y*. It is computed as the covariance of the two variables divided by the product of their standard deviations.) between corresponding elements of the empirical (and the simulated) covariance matrix, the statistics of the dynamic functional connectivity (DFC) can instead be evaluated through the Kolmogorov–Smirnov distance (Nonparametric Kolmogorov–Smirnov statistics provide a distance either between an empirical and a reference cumulative density function or between two probability distributions.) of the related empirical (and simulated) distributions of covariance matrix elements on a sliding time window basis. Note that, lately, this sliding window technique has been challenged by a hidden Markov model (A hidden Markov model represents the simplest dynamic Bayesian network. It is a statistical model of a system with Markovian state transitions between unobservable (hidden) states.) approach which overcomes some of the drawbacks of the former method [100–102]. Concerning the interrelation of the structural and functional connectivity, the work of the Deco group, as discussed above, demonstrated that, by carefully constraining global mean-field brain models with structural connectivity data and fitting the model parameters employing corresponding dynamic functional connectivity data, phenomenological models of causal brain dynamics (Dynamic causal models adapt nonlinear state-space models to data and test their evidence employing Bayesian inference.) [20] can be constructed. Such models yield insight into mechanisms by which the brain generates structured, large-scale activity patterns from spontaneous activities in RSNs while respecting empirical knowledge about structural and functional connectivities between distant brain areas as provided by functional neuroimaging.

Using a whole-brain computational network model, Glomb et al. [103] applied a sliding window approach, though employing a rather long time window, to relate temporal dynamics of RSNs to global modulations in BOLD variance. The authors demonstrated that spatiotemporal fluctuations in FC and BOLD can be described as fluctuations around an average stationary FC structure. In a related study, Glomb et al. [17] further elaborated this idea by combining dimensionality reduction via tensor decomposition with a mean-field model (MFM) [104] generating stationary network dynamics. The model has been shown to explain grand average resting-state FCs. However, such average FCs summarize correlated spatial activity distributions but do not reveal their temporal dynamics. In a two-step approach, first, spatiotemporal data arrays have been decomposed, employing tensor decomposition methods, into sets of brain regions, called communities, with similar temporal dynamics. Their related time courses are assessed by an overlapping sliding window technique and could be grouped into four distinct communities resembling well-known RSNs. Second, data were simulated with this stationary MFM constrained by results from diffusion tensor imaging (DTI) and fiber tracking. The spatiotemporal structure of the network then results solely from fluctuations around a mean FC pattern. More importantly, the four distinct RSNs emerged from this stationary MFM if the network nodes were coupled according to the model-based EC. The method is rather generative as it only needs weak assumptions about the underlying data, thus is generally applicable to resting-state data and task-based data from arbitrary subject populations. In a related study, Glomb et al. [21] compared the DTI-based SC with the model-based effective connectivity (EC) using whole-brain computational modeling of the spatiotemporal dynamics of FC evaluated on a sliding window basis. The authors discussed the way node connectivity affects the fitting of simulated to empirical patterns. The resulting tensors are decomposed into a weighted set of communities, whose nodes share similar time courses. Some of these communities resemble known RSNs such as the DMN, whose fluctuations have been linked to cognitive function. Similarity between simulated and empirical spatiotemporal dynamics of ROIs was especially pronounced whenever model nodes were connected by EC rather than SC. Thus, networks of Stuart–Landau oscillators with simple attractor dynamics can reproduce empirical spatiotemporal connectivity dynamics if constrained by EC rather than SC. A recent review of Cabral et al. [16] discussed different computational resting-state models, which almost all try to explain how a rich repertoire of functional connectivity states can emerge from a single static structural connectome. In the future, it will be of interest to extend these computational models to task-based settings and to also consider faster neural processes like those measured by EEG or MEG.

The potential of computational connectomics for general inference and integration of neurophysiological knowledge, complementing empirical functional neuroimaging studies, has been further demonstrated by the work of Schirner et al. [105]. The authors integrated individual SC and FC data with neuronal population dynamics to infer neurophysiological properties on multiple scales. In their study, EEG was used to record electrical potentials at the scalp surface of individuals. These source activities were integrated into an individual-specific model, which simulated brain activity distributions and predicted person-specific fMRI time series and spatial topologies of RSN activities. In addition, neurophysiological mechanisms underlying several experimental observations from various functional imaging modalities could be successfully predicted. Whole-brain computational modeling can also answer questions concerning the reproducibility and consistency of resting-state fMRI. Donnelly-Kehoe et al. [106] demonstrated that the estimation of parameters, which describe the dynamical regime of the Hopf model (It denotes a model of a dynamic system which exhibits a critical point in the phase space where its stability switches and a periodic solution arises.) [2], becomes consistent after a scanning time of around 20 minutes. This suggests that such a scanning duration is sufficient to capture subject-specific brain dynamics. Also, such nonlinear computational models are capable of quantifying EC on a whole-brain level [107]. By gradually modifying the strength of structural connections, the authors could improve the correspondence between FC predicted by their model and empirical FC observed in fMRI. This measure should therefore better characterize the activation flow between brain regions and can extend the structure-based connectivity measure, derived from DTI.

### 4.4. Impact of the Structure on Function in Computational Connectomics Models

An as yet unresolved issue concerns the dependence of the SC-FC relationship on either specific topological features of the network or the computational models used to describe the network dynamics. Over the last decade, a couple of studies, designing stationary and nonstationary dynamical network models, focused on these important issues.

By employing a simple epidemiological model, Chen and Wang [60] built a dynamic susceptible-infected-susceptible (SIS) network [108, 109], focusing on the shortest path to predict resting-state FC from SC. The model could predict FC between directly and indirectly connected structural network nodes and outperformed DMF models in predicting FC from SC. Considering a more complex approach, Robinson [110] introduced propagator theory (A propagator represents a special Green's function, which characterizes the probability of propagation of a particle or wave from location *x* to location *y*. The exact form of the propagator depends on the equation of motion with its related initial or boundary conditions.) to relate anatomical connections to functional interactions, where neural interactions allegorically resembled properties of photon scattering on atoms, as observed in standard quantum mechanics. This Green's function-based model also accounts for excitatory and inhibitory connections, multiple structures and populations, asymmetries, time delays, and measurement effects.

In another early study to elucidate the structure-function relationship in more detail, Deco et al. [111] devised a brain model of Ising spin dynamics constrained by a neuroanatomical connectivity as obtained from DTI/DSI data. The model, which describes stationary dynamics, exhibited multiple attractors, whose underlying attractor landscape could be explored analytically. They showed that the entropy of the attractors directly measures the computational capabilities of the modeled brain network, thus pointing to a scale-free (A scale-free network exhibits a degree distribution that, at least asymptotically, follows a power law.) architecture. Note that entropy measures the number of possible network configurations; hence, a scale-free network maximizes the system's entropy. However, recently, strictly scale-free networks have been shown to be rare putting in jeopardy statistical interpretations of such networks [112]. It has been shown that log-normal distributions fit degree distributions often better than power laws, thus demanding alternative theoretical explanations. A similar spirit, predicting SC from FC, has also been considered in the study of Deco et al. [104]. The authors used diffusion spectrum imaging (DSI) to map structural measures of connectivity, but note that interhemispheric connections are usually inhibitory and are hard to map. The study proposed a dynamic MFM operating near a critical point in the phase space where state transitions occur spontaneously. They iteratively optimized the matrix of SCs based on a matrix of FCs and observed that the addition of a small number of anatomical couplings, primarily transhemispheric connections, improved the predicted SCs dramatically, even though DTI has its limitations in modeling long-range connections [37].

While these modeling studies rely on stationary dynamics, Messè et al. [113] considered the relative contributions of stationary and nonstationary dynamics (A time series is stationary if all its statistical moments are time independent. Wide-sense stationarity only asks for time independence of the first two statistical moments, and nonstationarity means just the contrary [114].) to the structure-function relationship. The authors compared FCs of RSNs with computational models of increasing complexity while manipulating the models' anatomical connectivity. Their results suggest three contributions to FC in RSNs based on a scaffold of anatomical connections, a stationary dynamical regime constrained by the underlying SC, and additional stationary and nonstationary dynamics not directly related to anatomy. Most importantly, the last component was estimated to contribute 65% to the observed variance of FC pointing to the need for nonstationary dynamic computational brain models. The study corroborated the preference for simple models of stationary dynamics and emphasized the decisive role of transhemispheric couplings, which are often difficult to reconstruct in white matter tractography [37]. Messé et al. [72] also further considered the issue of how topological features influence dynamic models and designed a dynamic susceptible-excited-refractory (SER) model with excitable units and analyzed the influence of network modularity on FC. Their results were compared to a FitzHugh–Nagumo model (The FitzHugh–Nagumo model, sometimes also called Bonhoeffer–van der Pol oscillator, represents a relaxation oscillator and describes a prototype of an excitable system, which shows spike generation above a certain threshold.) [115] as an alternative model of excitable systems. Differences between the models arose from different time limits for integrating coactivations to deduce “instantaneous” FCs, thus providing a clear distinction between coactivation and sequential activation and thereby corroborating the importance of the modular structure of the network. In a subsequent paper, Messé et al. [71] elaborated further on the topological network features which shape FC in their dynamic SER model. The authors presented an analytical framework, based on discrete excitable units, to estimate the contribution of topological elements to the coactivation of the nodes in their model network. They compared their analytic predictions with numerical simulations of several artificial networks and concluded that their framework provides a first step towards a mechanistic understanding of the contributions of the network topology to brain dynamics.

## 5. Machine Learning Approaches

Machine learning approaches were first applied to fMRI datasets to deduce signal components in a purely data-driven fashion. Such explorative techniques allow to detect spatially segregated regions in the brain, associated with individual functions [116, 117]. These blindly identified source components can then help to define nodes in graphical models of brain networks and set the spatial layout for brain connectivity. Also, such blind source separation techniques were applied to EEG recordings in order to identify relevant source signals and to separate them from artifacts in the data [118–120]. Like in fMRI, such intrinsic components can be used to define nodes in functional networks and provide a data-driven perspective on brain connectivity in EEG studies [121].

Exploratory matrix factorization (EMF) techniques were employed mostly, whereby any data matrix **X** ∈ *ℝ*^*N*×*M*^ contains spatial fMRI maps in its *M* columns at *N* subsequent time points *t*_*n*_. This data matrix is decomposed into two factor matrices according to **X** ≈ **WH**, whereby **W** ∈ *ℝ*^*N*×*K*^ and **H** ∈ *ℝ*^*K*×*M*^. Here, *K* denotes a generally unknown inner dimension, which can be estimated with model order selection techniques [122]. Such decomposition needs additional constraints to yield unique answers. Depending on the form of the constraints, various decomposition techniques (see Appendix B) result as follows:

- Factors should form orthogonal matrices, yielding principal component analysis (PCA)
- Factor matrix **H** should contain statistically independent spatial or temporal components (sICA and tICA)
- Factor matrix **W** should yield a sparse encoding (SCA)
- Both factor matrices should have only nonnegative entries, given the entries of the data matrix are nonnegative exclusively, yielding nonnegative matrix factorization (NMF)
- Matrix **H** should contain intrinsic modes, which represent pure oscillations yet with time-varying amplitude and local frequency, yielding empirical mode decomposition (EMD)

Such studies were employed to deduce functionally connected brain networks in a purely data-driven way, most commonly by a combination of PCA and ICA [123]. Although the number of independent spatial or temporal components is generally unknown, investigations showed a high consistency of the extracted functional networks across subjects and conditions. Since structural constraints are yet to be included, structure-function relationships have not yet been considered so far with such techniques. Considering regularized ICA, comprehensive studies of the choice of hyperparameters and their impact on the results are still not completely explored. Some attempts have been undertaken to combine cICA with optimization techniques.

Over the last decade, exploratory analysis techniques have placed a new focus on trying to better understand brain dynamics. Dynamic functional connectivity patterns (also denoted as the chronnectome [124]) have been studied with EMF techniques by employing a sliding window approach, whereby a considerable overlap of the individual time segments was allowed. The length of the chosen time window determines the time scale of the slowest fluctuations that can be studied. Most commonly, time courses were then linearly correlated to generate temporal sequences of related connectivity matrices. Such sequences demonstrate the temporal variability of functional connections between identified brain networks, notably those investigated under resting-state conditions. The main purpose of such studies was to quantify the impact of spontaneous BOLD fluctuations on the temporal dynamics of FCs. The investigations demonstrated that transiently synchronized subnetworks with coherent spatial patterns drive the dynamics of large-scale functional networks in the resting brain. These results gave rise to the application of state-space models, whereby network states were represented by their covariance matrices [125, 126]. Predominantly, hidden Markov models (HMMs) have been employed to describe the underlying network dynamics. As an alternative to HMMs, Bayesian probabilistic models can also learn latent states and represent dFC networks and their temporal evolution as well as transition probabilities between these states.

State-space models assume stationary dynamics, an assumption that has been placed in question by several studies. These studies assigned part of the temporal variability of dFCs not to noise contributions but rather to their nonstationary nature [127, 128]. The latter can be characterized through EMF approaches combined with sliding window techniques and Pearson correlation of voxel time series. Clustering such dFC states results in a small number of prototypical FC patterns, which, in turn, lead to discrete brain states [127, 129]. Alterations of such brain states with various diseases were naturally of interest as well.

### 5.1. Static Functional Connectivity

Over the last two decades, aside from large-scale computational modeling of functional brain connectivity and dynamics, data-driven machine learning approaches have also been employed to analyze functional neuroimaging data and to track the dynamics of FCs. In various cases, methods from machine learning, such as exploratory matrix factorization (EMF) techniques, can be applied, where voxel-wise univariate evaluations are not appropriate [116, 130]. Blind source separation (BSS) techniques [131–133] refer to data-driven, unsupervised machine learning techniques for feature extraction based on EMF, which are applied in biomedicine and neuroinformatics. The underlying idea of these techniques is to search for a linear mixture of base components, which characterize the observed data. Especially in the absence of stimulus-driven tasks, like in resting-state fMRI [116] or in resting-state EEG [121], such exploratory techniques have proven to be a promising alternative to atlas-based definitions of brain networks. Most notably, principal component analysis (PCA) and independent component analysis (ICA) (While PCA extracts components with maximal variance from the data, ICA applies a stronger condition and maximizes for statistical independent components [133]) [134] are frequently employed to analyze biomedical and neuroimaging datasets, especially EEG and fMRI data [117, 118]. While most studies on brain connectivity still rely on atlas-based definitions of graph nodes in brain networks, simulations have shown that data-driven derivations of such nodes with ICA can be beneficial for graphical analysis [36].

**Figure 5:**
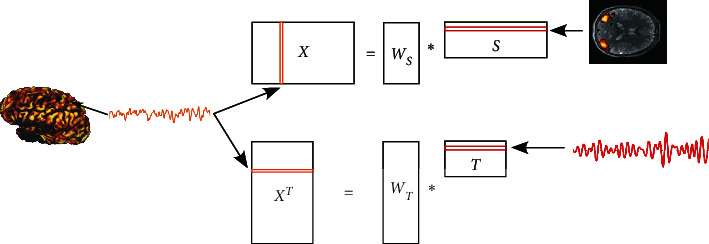
The principle of ICA is illustrated. As the input, a voxel time series is considered and indicated as an orange stripe in the related data matrices. The decomposition is either done to obtain independent spatial maps in component matrix *S* or to obtain independent component time series contained in the rows of component matrix *T*.

ICA is intrinsically a multivariate approach, and hence, each independent component (IC) groups brain activity into similar response patterns thereby providing a natural measure of functional connectivity (FC). ICA comes in two flavors extracting either spatially (sICA) or temporally (tICA) independent component maps. The basic principle of spatial and temporal ICA is illustrated in Figure 5, but these studies predominantly rely on sICA due to the abundance of requisite data samples. Spatial ICA has been applied first to fMRI datasets by McKeown et al. [117], while tICA followed shortly afterwards [135]. Also, few studies dealing with spatiotemporal ICA have been performed [136–141]. These various modes of ICA all share the limitation that the user has to identify the underlying sources. To resolve this issue, constrained ICA [142, 143] or ICA with reference [144, 145] has been proposed to extract one or more ICs, which are as similar as possible to given reference signals. Thus, a priori information of the desired sources is used to form constraints in either the spatial or temporal domain. This approach has also been extended to the spatiotemporal domain. The constrained stICA algorithm searches for maximally independent sources that correspond to constraints in both spatial and temporal domains [137]. This also exhibits improved performance for the analysis of fMRI datasets.

Early work studied cortical functional connectivity (FC) in a seed-based approach, where the time course of any chosen seed voxel was correlated with the time courses of all other voxels to reveal two-point correlations of cortical activity in response to external stimuli and task requirements. Such seed-based correlation analysis (sCA) studies are generally biased by the choice of the seed region. Several studies elaborated on the difference between sCA- and ICA-derived measures of FC. Already a decade ago, Joel et al. [146] concluded that seed-based FC measures are the sum of ICA-based measures both within and between network connectivities. Very recently, Wu et al. [147] proved a mathematical equivalence between sCA and a connectivity-matrix enhanced ICA (cmICA). However, they also noted conceptual differences, which lead to different information captured by both techniques and which they exemplified in examining whole-brain rsFC at the voxel resolution in schizophrenic patients and healthy controls. FC is reduced over the entire brain, whereby the connectivity not only between networks but also within network hubs is affected. In the resting state, decreasing FC was in both groups which also strongly related to aging in both groups.

An overview of exploratory ICA, applied to deduce the functional connectivity from the fMRI data, was given by Calhoun et al. [148]. The authors discussed ICA in the spatial or temporal domain related to task and transiently task-related paradigms as well as physiology-related signals, the analysis of multisubject fMRI data, the incorporation of a priori information, and the analysis of complex-valued fMRI data. While most studies only take the magnitude of the fMRI signal into account, it was shown that the phase information of complex-valued fMRI has the potential to increase the sensitivity of ICA [149]. More recently, it was demonstrated that spatial resting-state networks observed in fMRI could also be found in high-temporal-resolution EEG data using ICA [150]. In their study, Sockeel et al. [150] could observe several overlapping EEG and fMRI networks in motor, premotor, sensory, frontal, and parietal areas.

Studies of FC in the resting state started only later when the work of McKeown et al. [151] suggested that this could be possible. As Calhoun and Adali noted in a focused survey [153], ICA offered, and still offers, essential methodological tools to study the functional connectivity of brain networks not only in single subjects but also across whole groups. The focus then shifted from task-related paradigms to the study of resting-state networks (RSN) and, most importantly, their differences in the diseased brain. It has been shown that even in the absence of a stimulus-driven task, a number of brain networks, such as the default mode network (DMN), could be observed at rest [5, 123] and successfully reconstructed with ICA [130]. The major use of the DMN is made in fMRI studies of brain disorders. In an early investigation, Esposito et al. [154] considered the cognitive load modulation of group-level ICA-based fMRI responses. They suggested that the high variability of the default mode pattern may link the DMN as a whole to cognition and may more directly support the use of the ICA model for evaluating cognitive decline in brain disorders.

An early study on rsFC with sICA was published in 2004 [155]. The authors identified many of the known RSNs, and their ICs showed an extremely high degree of consistency in spatial, temporal, and frequency parameters within and between subjects. These results were discussed in relation to the functional relevance of fluctuations of neural activity in the resting state. Such high spatial consistency of cortical functional networks across subjects was also found by Beckmann et al. [130], who applied probabilistic ICA and discussed the role of this exploratory technique can take in scientific investigations into the spatiotemporal structure of RSNs. The exploratory nature of ICA was also stressed by Rajapakse et al. [156] in contrast to covariance-based methods such as principal component analysis (PCA) and structural equation modeling (SEM), where SEM is employed to automatically find the connectivity structure among elements in independent components. However, their hybrid ICA/SEM approach was restricted to task-related fMRI paradigms. Meanwhile, numerous studies have been published based on exploratory, data-driven fMRI analyses, which established ICA in its many flavors as a standard technique to analyze fMRI datasets with respect to FC, most notably of RSNs and the DMN. Also, a recent claim by Daubechies et al. [157] that the ICA algorithms Infomax and FastICA (The Infomax algorithm is based on the minimization of mutual information between estimated components, while FastICA follows the idea of maximization of non-Gaussianity of components [133]. Both algorithms have shown to be reliable for the estimation of independent brain networks [152].), which are widely used for fMRI analysis and which are based on different principles like those of entropy or cumulant expansion, select for sparsity rather than independence has been refuted by Calhoun et al. [158]. The latter authors claimed that the ICA algorithms are indeed doing what they are designed to do, which is to identify maximally statistically independent sources.

Because of scaling and permutation indeterminacies of ICA, group inferences from multisubject studies turned out to be challenging. Several attempts have been considered to resolve this issue [159–163]. The most widely used approach is based on the gICA algorithm provided in the GIFT toolbox (http://mialab.mrn.org/software/gift/). A large-scale study [164], encompassing 603 healthy adolescents and adults, employed gICA to establish a multivariate analytic approach and applied it to the study of RSNs. The latter were identified and evaluated in terms of three primary outcome measures: time-course spectral power, spatial map intensity, and functional network connectivity. The study considered the impact of age and gender on resting-state connectivity patterns. The results revealed robust effects and suggested that the established analysis pipeline could form a useful baseline for investigations of human brain networks. Recently, we proposed a hybrid cICA-EMD approach, where a bidimensional ensemble empirical mode decomposition technique based on Green's functions in tension (GiT-BEEMD) was used to create reference signals for a constrained ICA [165, 166]. The idea of this technique is to decompose a signal into its underlying intrinsic frequency compartments [56], reflecting frequency-specific aspects of the latter. The natural ordering of the intrinsic modes (IMs) extracted with GiT-BEEMD provides an immediate assignment of ICs, extracted with cICA, across a group of subjects. Results of both methods are in good agreement. However, the consistency of identified functional networks across a group of subjects is higher for the hybrid cICA-EMD approach. Still, one of the problems of cICA algorithms is the choice of hyperparameters such as the threshold for similarity measures or the accuracy of a priori information. Shi et al. [167] recently tackled such problems by combining cICA with multiobjective optimization, where the inequality constraint of traditional cICA is transformed into the objective optimization function of constrained stICA, and both temporal and spatial prior information are included simultaneously. The algorithm apparently avoids the threshold parameter selection problem, shows an improved source recovery ability, and reduces the requirements on the accuracy of prior information.

### 5.2. Temporal Dynamics of Functional Connectivity

**Figure 6:**
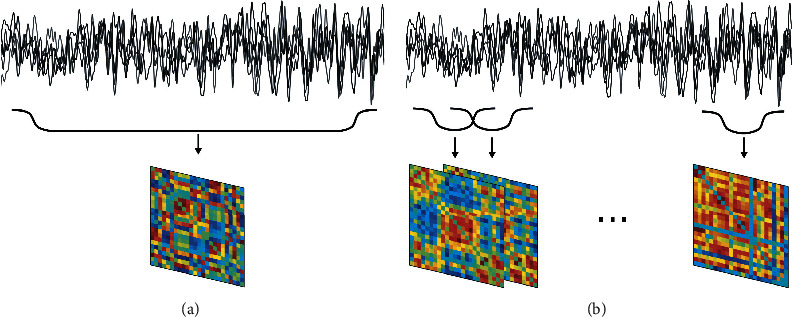
Illustration of the concepts used to derive the static and dynamic functional connectivity (dFC). (a) An example of various activity time courses and their related static connectivity matrix, which is deduced from the complete session. (b) The same set of local activity time courses and their related connectivity matrices of the respective segments of the activity time courses.

The explorative techniques discussed so far all concern investigations of static functional connectivity. However, as we have seen from investigations into computational brain dynamics, the brain is operating in a metastable state close to a critical point, where spontaneous fluctuations play a decisive role in determining the inherent dynamics of brain networks. Such fluctuations emerge on time scales ranging from milliseconds to minutes but have largely been ignored in most recent investigations involving data-driven techniques. Still, over the last decade, a paradigm shift has occurred in functional connectivity studies towards the focus on the temporal variations in FC patterns [168]. Most studies on dFC employ a sliding window technique, such as illustrated in Figure 6. Instead of computing FC across the whole time span of a session, dFC accounts for the variability of connectivity within a session by assessing FC on (possibly overlapping) segments in the time domain, but it also has been shown that this technique is not without problems in itself [169–171]. Also, a sound statistical analysis of such studies is mostly lacking, thus casting doubts on the interpretations given to the results [172].

A number of studies considered the dFC of BOLD signals and their related spatial patterns based on sliding window correlations (SWCs). With this new focus, Chang and Glover [173] investigated the dynamics of resting-state connectivity patterns during a single fMRI scan. They performed a time-frequency coherence analysis based on the wavelet transform and employed a sliding window correlation procedure to demonstrate time-varying connectivity patterns between several brain regions. The authors noted that such coherence and phase variability might be the result of residual noise rather than resulting from modulations of the cognitive state. In a similar study, Kang et al. [174] thoroughly investigated the temporal FC of spontaneous BOLD signals derived from RSNs with fMRI. RSNs were identified using a seed-based voxel-wise correlation analysis by calculating correlations between representative time courses of certain predefined regions and all other voxels of interest. A subsequent variable parameter regression model, combined with a Kalman filter for optimal model parameter estimation, was applied to identify dynamic interactions between the identified RSNs. The results revealed that functional interactions within and between RSNs showed indeed time-varying properties. Furthermore, the spatial pattern of dynamic connectivity maps obtained from adjacent time points exhibited a remarkable similarity. Employing ultrahigh field fMRI, Allan et al. [175] further investigated the contribution of spontaneous BOLD events to the temporal dynamics of FC and suggested that spontaneous fluctuations of BOLD signals drive the dynamics of large-scale functional networks commonly detected by seed-based correlation and ICA. These suggestions were based on observations that spontaneous BOLD signal fluctuations contribute significantly to network connectivity estimates but do not always encompass whole networks or nodes. Rather, clusters of coherently active voxels forming transiently synchronized subnetworks resulted. Furthermore, tasks can significantly alter the number of localized spontaneous BOLD signals. From these observations, the picture emerged that large-scale networks are manifestations of smaller, transiently synchronizing subnetworks of voxels whose coherent activity dynamics give rise to spontaneous BOLD signals. Recent fMRI studies demonstrated that the dynamics of spontaneous brain activities and the dynamics of their functional interconnections show similar spatial patterns suggesting they are associated to each other. Thus, Fu et al. [176] characterized local BOLD dynamics and dFC in the resting state and studied their interregional associations. Again, dFCs were estimated employing the aforementioned sliding window correlation technique, and BOLD dynamics were quantified via the temporal variability of the BOLD signal. BOLD dynamics and dFC indeed exhibited similar spatial patterns, and they were significantly associated across brain regions. Interestingly, intra- and internetwork connectivities were either positively or negatively correlated with the BOLD signal and exhibited spatially heterogeneous patterns. These associations either conveyed related or distinct information pointing towards underlying mechanisms involved in the coordination and coevolution of brain activity.

Though, in the first decade of the new millennium, an increasing number of dynamic FC studies have appeared, Thompson et al. [177] noted that only few investigations used small enough time scales to infer single subjects' behaviors. While studying the interaction between the DMN and task-positive networks within a psychomotor vigilance task, they evaluated correlations between the two networks' signals within a time window of 12.3s, centered at each peristimulus time interval. In addition, correlations were also computed within entire resting-state fMRI runs from the same subjects. These correlation measures were compared to time lags of response signals, both intra- and interindividually. Generally, significant anticorrelation was related to shorter response time lags interindividually, while single subjects showed this behavior only 4⟶8s before the detected target. Hence, studies of the relation between functional networks and behavior are valid only on short time scales and need to take into consideration the inter-as well as intraindividual variability. These early findings of studies devoted to dynamic FC were summarized and evaluated by Hutchison et al. [178].

Considering that variability of neural activity is a hallmark of intrinsic connectivity networks identified by rs-fMRI, Jones at al. [128] hypothesized that the variability, rather than representing noise, is also related to the nonstationary nature of those networks, switching between various connectivity states over time. The authors noted that this variability has hampered efforts to define a robust metric of connectivity that could be used as a biomarker for neurologic illness. Employing gICA and a large cohort of 892 older subjects, 68 functional ROIs were defined, and, for each subject, a dynamic graphical representation of brain connectivity was constructed within a sliding window approach to demonstrate the nonstationary nature of the brain's modular organization. When comparing dwell time in strong subnetworks of the DMN of a group of subjects suffering from Alzheimer's dementia with a healthy control group, it was concluded that connectivity differences between these groups are due to dwell time differences in DMN subnetwork configurations rather than steady-state connectivity.

Afterwards, in a seminal paper, Allen et al. [127] studied resting-state FC dynamics of the entire brain based on spatial ICA, Pearson correlation within sliding time windows, and *k*-means clustering of correlation matrices (*k*-means clustering aggregates a number observations into groups based on predefined similarity measures. Groups can then be represented by prototypical observations like the mean inside a group.) within such windows. The study encompassed a large sample of 405 young adults and was based on employing gICA for extracting spatially independent connectivity patterns across the subject cohort. This study identified particularly flexible connections between specific brain regions which, therefore, cannot be considered separate and antagonistic entities. More importantly, however, the study introduced and identified dynamic FC states which differed from stationary connectivity patterns, thus challenging the common descriptions of static interactions between large-scale networks. Their findings also suggested the need to track functional connectivity dynamics and to exploit the role of these dynamical processes for a better understanding of behavioral shifts and adaptive processes. Recently, Goldhacker et al. [129] added another twist to such sliding window investigations of dynamic functional connectivity (dFC) of the resting state. The authors introduced frequency-resolved dFC by means of multivariate empirical mode decomposition (MEMD) [179–181] followed up by filter-bank investigations. Entire voxel time courses were decomposed with MEMD into intrinsic modes (IMs). Next, sliding window connectivity matrices were established for every IM separately, thus reflecting the temporal development of connectivity patterns established on various intrinsic time scales. As IMs are naturally ordered according to their characteristic frequencies, the resulting connectivity matrices followed this ordering. When *k*-means clustering was applied to this vast amount of frequency-resolved connectivity matrices, each cluster centroid represented a connectivity state at a specific time scale determined by the period of the intrinsic oscillation of the related IM. It was observed that the structure of such connectivity states was persistent across several time scales and even became more pronounced with an increasing period of the intrinsic oscillation. To quantify the similarity across frequency scales, a Pearson correlation similarity measure for dFC states is introduced. However, scale stability changed with the number of extracted clusters and dropped off between *k* = 4 and *k* = 5 extracted connectivity states. This finding was corroborated by null models, simulations, theoretical considerations, filter-banks, and scale-adjusted windows. These filter-bank studies showed that filter design is more delicate in the rs-fMRI than in the simulated case. The study presented the first evidence indicating that connectivity states are both a multivariate and a multiscale phenomenon. Besides offering a baseline for further frequency-resolved dFC research, the authors demonstrated the use of scale stability as a possible quality criterion for connectivity states and the related model selection problem.

Soon after the seminal work of Allen et al. [127], dFC studies were applied to fMRI investigations of subjects suffering from brain disorders. Damaraju et al. [182] studied schizophrenia, which is a psychotic disorder characterized by functional dysconnectivity or abnormal integration between distant brain regions. They evaluated static and dynamic functional connectivity networks of a large cohort of schizophrenic patients and healthy controls. While static correlations encompassed time series of 5.4min length, dFC was determined using a sliding window technique with a window length of 44s. *k*-means clustering then resulted in five discrete functional connectivity states, so-called brain states. Especially, dFC states showed characteristic differences in time-varying connectivity patterns between schizophrenic patients and healthy controls that could not be observed with a static correlation analysis alone. Considering these concerted attempts to clarify the dynamical nature of FC, Calhoun et al. [124] went on to coin the term chronnectome for these endeavors. The term is intended to denote metrics which provide a dynamic view on functional couplings underlying temporally fluctuating and spatially evolving brain connectivity patterns. The authors focused their review on their own work, developing EMF techniques in an attempt to solve BSS problems and also discussed a number of methodological directions. A recent application to mild cognitive impairment detection was reported by Yan et al. [183]. The authors considered a deep learning ansatz and devised a fully connected bidirectional long short-term memory (LSTM) (Long short-term memory neural networks (LSTMs) are a class of artificial recurrent neural networks, which are able to effectively detect long-term relations in sequential data structures [162].) network (Full-BiLSTM) to effectively learn periodic brain status changes.

In a timely review on the subject, Preti et al. [184] provided a comprehensive description of proposed dFC approaches, pointed at future directions of dFC research pointing out advantages and pitfalls. The subject has also been reviewed by Karahanoğlu and Ville [185]. Yet, another review by Betzel and Bassett [186] focused on the multiscale (ms) aspect of modern brain connectivity studies. The discussion was separated into ms-topological structures, ranging from individual nodes to complete networks, ms-temporal structures, spanning all available time scales of measurements, and ms-spatial structure, referring to the granularity at which its nodes and edges were defined. The authors reviewed empirical evidence for such structures and discussed network-based methodological approaches to reveal these structures on their respective scales.

Finally, a small number of studies critically discussed a common methodology to study dFC concerning sliding window techniques in the absence of any solid statistical analysis. While a number of dFC studies were carried out in the last decade, Hindriks et al. [172] pointed out that their statistical analysis was either not always carried out properly or was even omitted in some cases. The authors described the appropriate statistical tests for dFC, assessed the performance of dFC measures, and illustrated the proposed methodology with a study of spontaneous BOLD signal fluctuations in rs-fMRI recordings. Sliding window correlations were considered predominantly to cope with the intrinsically nonstationary nature of such correlations. Nonlinear correlation measures were also considered. The authors concluded from simulations that, with resting-state sessions of 10min duration or less, dFC cannot be detected using sliding window correlations. Applying session averaging or subject averaging, most functional connections could be shown to be dynamic. For the first time, this study pointed out the necessity of a sound statistical analysis of fMRI investigations of dFC and pointed out possible statistical pitfalls in such studies.

As most studies of dFC capture the dynamics via a sliding window technique, Shakil et al. [171] noted that, in the absence of any gold standard, sliding window correlations can be problematic for the analysis of resting-state data. The authors devised simulated networks to examine the impact of window length, window offset, window type, noise, filtering, and sampling rate on the performance of sliding window correlational analysis. Activity time courses of all node pairs of each simulated network were correlated and then grouped together using *k*-means clustering. It could be shown that resulting brain states and state transitions strongly depended on window length and offset and, to a lesser extent, on noise and filtering parameters. Also, tapered windows were less sensitive to state transitions than rectangular windows. Clustering only yielded reliable estimates of state transitions if the window size matched the length of the state duration. Similar concerns about the reliability of a sliding window correlation technique were addressed by Kudela et al. [170] who combined a multivariate linear process bootstrap [187] (MLPB) method and a sliding window technique to assess the uncertainty in a dynamic FC estimate by providing its confidence bands. This additional statistical evaluation should separate the true signal from spurious fluctuations generated by noise. Yet, another way around the sliding window dilemma was proposed by Andersen et al. [169], who suggested a Bayesian approach to dFC (A Bayesian approach allows to incorporate previous knowledge about a quantity in the form of a prior into an estimation, deduced from a posterior probability distribution.), where covariances varied smoothly over time and the related brain states were represented by spatially sparse components. This approach is based on the idea that brain functions can be represented by a small number of cognitive components [188]. Based on a simple classification task, the authors claimed that their model better captures the underlying structure.

**Figure 7:**
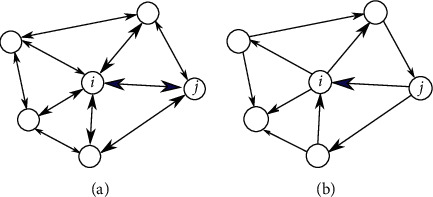
Correlation-based functional connectivity describes the temporal coherence of activity profiles in two brain regions *i* and *j* and therefore yields undirected graphical representations of brain networks (a). On the contrary, directed connectivity measures such as Granger causality provide a data-driven perspective on potentially causal dependencies among brain regions, i.e., if one region *i* drives region *j*, or vice versa (b).

Due to the low signal-to-noise ratio (SNR) of the BOLD signal and the massive amount of data produced in any resting-state fMRI investigation, Choe et al. [189] investigated the reliability and robustness of summary measures based on sliding window correlations (SWCs), tapered sliding window techniques, and dynamic conditional correlation (DCC) methods. Such DCC approaches extend the classical correlation measures by additionally estimating conditional correlations. They applied these methods to two large public data repositories (Multimodal MRI Reproducibility Resource and Human Connectome Project) and assessed two categories of dFC summary measures, namely, basic summary statistics, such as mean and variance of dFC across time, and summary measures derived from brain states, such as the dwell time. Though DCC methods outperformed SWCs with respect to summary statistics, the reliability of brain state-derived measures was low. Especially, DCC-derived dFC variances were significantly more reliable than those following from nonparametric estimation methods. These findings show that dFC variance should form an important ingredient to any dFC-derived summary measure. With a similar interest, Thompson et al. [190] studied time-varying connectivity (TVC) and developed a Python package, called *tvc*_*benchmarker* (https://github.com/wiheto/tvc_benchmarker), providing four simulations, and used them to test five different methods to estimate their ability to track the dynamics of activity covariances over time: sliding window, tapered sliding window, multiplication of temporal derivatives, spatial distance, and jackknife correlation. All methods revealed positive correlations but with strongly varying magnitudes. This tool can help scientists to evaluate their analysis methodologies in the face of any missing ground truth concerning dFC in the brain. Aside from fMRI investigations of dFC, recently, functional techniques operating on much shorter time scales have been considered. Granger causality (GC) measures directional dependence between time series, most importantly, activity time series from different brain areas such as illustrated in Figure 7. Early work on connectivity analysis based on EEG and MEG techniques is mainly concerned with alternative ways to detect interdependencies between activity time series.

Sato et al. [191] proposed partial directed coherence (PDC) [192] as a frequency-domain alternative to GC-based connectivity analysis of multisubject fMRI data. The authors employed multisubject bootstrapping and decomposed EEG-deduced connectivity data in the frequency domain, thus separating out artifact signals such as scanner noise, breathing mode, and heartbeat. If GC is estimated via vector autoregressive models (VARs), numerous parameters need to be estimated, which encompasses low accuracies with small datasets. Siggiridou and Kugiumtzis [193] proposed a restricted VAR model, which combined a modified backward-in-time selection (BTS) of lagged variables with a conditional Granger causality index (CGCI). The new method is applied to multichannel scalp EEG recordings of epileptic patients. The authors were able to track changes in brain connectivity before, during, and after epileptiform discharges with the proposed time-ordered VAR model. Simulations of high-dimensional, nonlinear systems with time series of varying lengths allowed them to favorably compare CGCIs obtained with other restricted or LASSO-constrained VAR models. Aside Granger causality (GC), conditional mutual information, also called transfer entropy (TE), offers an alternative way to study effective connectivity in the brain. TE reduces to GC for VAR models but generalizes GC to nonlinear processes. TE has been applied by Vicente et al. [194] to magnetoencephalography (MEG) recordings in a simple motor task. The authors demonstrated the superior detectability of causal relations deduced from MEG signals as TE is insensitive to signal cross-talk due to volume conduction. Multivariate vector autoregressive (MVAR) models are often used to estimate brain connectivity from EEG signal recordings. MVAR models have first been implemented by Antonacci et al. [195] on ANNs. The authors showed that stochastic gradient descent with *L*1-regularization, if applied during learning directly on the estimated weights, can efficiently cope with small datasets as well as regressor collinearity and provide accurate brain connectivity estimates. Subsequently, Antonacci et al. [196] extended their studies of network information processing with small available datasets acquired to investigate network physiology problems. They proposed a state-space (SS) variant of a VAR model regularized by the LASSO constraint and applied it to the analysis of the physiological network of brain and peripheral interactions probed in humans under different conditions of rest and mental stress. Their study corroborates the possibility to extract physiologically plausible patterns of interaction between the cardiovascular, respiratory, and brain wave amplitudes. Last but not least, Antonacci et al. [197] focused on the estimation of Granger causality (GC) in adverse conditions of small datasets or a very high number of time series. The authors showed that it is still possible to estimate GC in linear interaction time series and to reconstruct the underlying network structure if VAR models are combined with state-space models and partial conditioning on a subset of most informative variables [198].

The study of Nobukawa et al. [199] dealt with related electroencephalogram (EEG) data to analyze the continuously captured time-varying instantaneous phase synchronization between resting-state EEG potentials from different brain regions. For the first time, the temporal dynamics of phase synchronization was characterized using multiscale entropy, which quantifies the complexity of brain signal dynamics over multiple temporal scales. Comparing groups of healthy younger and older subjects, a region-specific enhanced complexity of temporal dynamics of phase synchronization was observed in older subjects in the *α*-band predominantly in frontal brain regions. Such altered complexity was not identified by a comparative phase synchronization approach such as phase lag index, which is defined by the consistency in the distribution of instantaneous phase differences. Surrogate analyses confirmed the deterministic origin of the temporal dynamics of phase synchronization in the neural network system. Phase-locking mechanisms were also the subject in the study conducted by Lee et al. [200]. By employing EEG recordings, they suggested that partial phase locking is the underlying concept of optimal functional connectivity during rest. The measurement of phase lag diversity enables us to quantify how far pharmacologically or pathologically perturbed connectivity deviates from its critical state, which could be used to identify various states of consciousness. In an effort to bridge the gap across different time scales in connectivity analysis, Wirsich et al. [201] proposed an integrative framework which combines FC observed in EEG and fMRI simultaneously. In their study, they employed a hybrid connectivity independent component analysis (connICA) [73] to search for spatially independent networks linked between the two modalities. Their study could reveal two robust hybrid components, one which is uniformly distributed across EEG frequencies, while the second one showed higher sensitivity to different EEG frequency bands. Their results suggest that some spatially independent FC patterns are coexpressed simultaneously in EEG and fMRI.

### 5.3. Hidden State-Space Models

**Figure 8:**
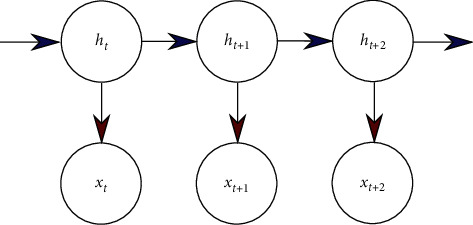
Illustration of a simple hidden Markov model (HMM). Such models are based on the idea that observable events **x**_*t*_ are in a causal relation with some underlying hidden events **h**_*t*_.

Given that spontaneous fluctuations of BOLD signals in RSNs reflect the dynamic organization of the resting brain, a number of studies employed state-space models, based on neural activity patterns or functional connectivity states, to characterize underlying dynamic brain mechanisms. Leonardi et al. [202] proposed a data-driven approach based on PCA to reveal latent coherent FC dynamics in a multisubject fMRI study. The study compared principal components of whole-brain dynamic FC patterns of multiple sclerosis patients with those derived from a healthy control group. In multiple sclerosis patients, they identified a network of altered connections centered on the DMN. Instead of decomposing whole-brain dynamics into eigenmodes, Eavani et al. [203] decomposed such subject-specific functional connectivity patterns into a temporal sequence of hidden states employing a hidden Markov model (HMM). The basic principle of a HMM is illustrated in Figure 8, and the general principle is outlined in Appendix C. In this study, states were represented by their unique covariance matrices reflecting the underlying whole-brain network. These covariance matrices were generated from a set of sparse basis networks, each reflecting a specific pattern of functional activity of selected regions of interest (ROIs). Hidden network states arose in this model from distinct variations in the strength with which different basis networks contributed to any specific hidden state. The model explained the functional activity as a dynamically changing combination of overlapping task-positive and task-negative basis networks.

In an effort to analyze dynamic transition patterns of functional brain states, Ou et al. [125] also investigated an HMM to characterize what they called functional connectome states. The study focused on a rs-fMRI dataset which encompasses posttraumatic stress disorder patients and normal controls. The study revealed that the brain only switches between a set of few brain states. Furthermore, two HMMs, one for patients and one for healthy controls, were constructed, and classification could be performed by examining which of the two HMMs can better describe the observed connectome state sequence. Rather than relying on HMMs alone, Taghia et al. [204] developed a Bayesian generative model within the framework of HMMs resulting in a dynamic variant of the static factor analysis model [205, 206]. In their study, Bayesian switching factor analysis (BSFA) learns latent states, representing dFC networks, and their temporal evolution, as well as transition probabilities between these states. Variational Bayes learning also allows us to estimate the number of latent states via Bayesian model selection, thereby preventing the development of overly complex models. Finally, the BSFA model was thoroughly tested on data extracted from the Human Connectome Project [207]. Though the HMM has been commonly used in modeling brain states, Shappell et al. [126] recently pointed out that HMMs assume the sojourn time (i.e., number of time points in a brain state) to be distributed geometrically. Otherwise, inaccurate estimates of the dwell time in any brain state might result. The authors proposed a hidden semi-Markov model, which explicitly models the mean waiting time distribution for each brain state. Application to task-based as well as resting-state data revealed the potential of mean waiting time, also called sojourn time, distributions for an understanding of healthy and diseased brain mechanisms. An interesting variant of a HMM that has not yet been applied to dFC data has been proposed by Kohlmorgen [208]. This approach considers the situation of a nonstationary dynamical system, which switches between a number of dynamically changing states. The model processes the data incrementally and does not need to learn internal parameters. It relies on an online variant of the Viterbi algorithm (A Viterbi algorithm generates the maximum likelihood estimate of the sequence of hidden states within an HMM.) and provides an online exploratory data segmentation and classification. This is achieved by tracking and segmenting changes of the underlying probability density in a sliding window approach.

Though much effort has been devoted to characterize resting-state dFC, its nature is still the subject of ongoing discussions. As neuronal populations coordinate their activities on specific time scales through phase coherence, Vidaurre et al. [209] employed HMMs to analyze magnetoencephalogram (MEG) data with respect to characterizing the dynamics in large-scale phase-coupled networks, which show coherent activity patterns. They could show that RSNs can be represented on short time scales by transient brain states, which are characterized by short-lived spatial patterns of phase coherence and oscillator strengths that in part resemble DMNs. Thus, functional specialization in the brain may transiently occur at various intrinsic time scales through large-scale phase coupling mechanisms. In a subsequent study, Vidaurre et al. [210] extended their HMM to the big data scenario. The amended HMM was able to infer robust and interpretable dFC across a set of data encompassing task-based and resting-state paradigms, recorded via either MEG or fMRI of thousands of subjects. Such tools are essential to make progress while taking advantage of huge data repositories provided by the Human Connectome Project or the UK Biobank initiative.

Although time-varying connectivity studies explored the nonstationary nature of the dynamical switching between discrete brain states, recent investigations in the field cast doubts on the nonstationarity assumption. Rather, these studies suggested that dFC might be attributed to sampling variability of static FC. This controversy led Liégeois et al. [211] to reanalyze the stationarity and statistical testing of dFC. They pointed out the relation of stationarity to ensemble statistics, while all FC measures in use are related to sample statistics. This fact broadens the space of stationary signals to include the important class of HMMs often employed to construct discrete brain states. That is to say that stationarity is not in contradiction to the concept of a steady state with a finite number of states between which the system switches dynamically. An in-depth discussion of issues related to statistical testing in case of FC was presented. Commonly used concepts such as phase randomization (PR) and autoregressive randomization (ARR) generate stationary, linear, and Gaussian distributed data, and this null hypothesis cannot be rejected by most subjects taken from the Human Connectome Project for testing. An immediate consequence is that, by applying such tests, statistical rejection can be caused by inherently nonlinear and nonnormally distributed data. Hence, nonlinear and/or non-Gaussian models may provide a better explanation of the dataset under study. The authors corroborate this in their study where 1st-order autoregressive (AR) models explained their data significantly better than static FC models. They show that such models better replicate empirically observed dynamic connectivity patterns in rs-fMRI data, which, in their opinion, could indicate a lack of discrete brain states. On the contrary, the HMM can capture aspects of the data that the AR model cannot capture, for example, that the transitions between networks organize hierarchically [212], or how the visits to these networks relate to the sleep cycle [213]. Therefore, AR and HMM models can reveal complementary aspects of brain dynamics. Issues related to statistical testing of dFC models have also been discussed by Khambhati et al. [214] who reviewed efforts to model dFC and related activity patterns and provided suggestions for a careful and accurate interpretation of dynamic graph architectures.

### 5.4. Machine Learning Approaches to the Structure-Function Relationship

As discussed above, aside from large-scale computational modeling of functional brain connectivity and dynamics, so-called computational connectomics, machine learning approaches, often based on EMF, have been used to explore, interpret, and classify functional connectivity patterns [215, 216], but up until now, only relatively few machine learning-based studies dealt explicitly with the structure-function relationship.

An early attempt has been made by Deligianni et al. [217] to quantify the prediction quality of FC based on known SC. In their first study, they used a canonical correlation analysis- (CCA-) based model to infer functional connectivity from the structural connection strength. By finding maximally correlated projections of two sets of variables (e.g., SC and FC), CCA can reveal hidden interrelationships between them. This mapping problem is characterized by its high dimensionality: when using *n* regions of interest (ROIs), defining the nodes of the structural/functional graph, one would need to infer *N*=*n*(*n* − 1)/2 functional connections from an equal amount of structural connections. To address this problem, the authors additionally used principal component analysis (PCA) for dimension reduction of the SC and FC data before applying CCA.

Another way to deal with high-dimensional problems offers the least absolute shrinkage and selective operator (LASSO), which was used in further studies of Deligianni et al. [74, 218–220]. This sparse linear regression model shrinks noisy and irrelevant connections to zero and also performs feature selection by linking each output variable with a subset of input variables [221]. At first, Deligianni et al. [218] used this model to directly infer FC from SC. Employing this model, they studied the impact of indirect structural connections between regions (see Figure 3), and a significant improvement of the prediction performance was shown when indirect connections up to the second order were added [218]. In subsequent investigations [219, 220], instead of trying to directly predict functional connectivity as the covariance matrix of mean time series in different ROIs, they used a parametrization based on a multivariate autoregressive model to describe the generative process of fMRI time series. In addition to this probabilistic framework, they relied on a randomized version of LASSO, which randomly incorporates different weights for the regularization, what makes the regression problem less dependent on the choice of parameters. Each structural connection can then be assigned with a probability to be selected for predicting a functional connection, making it possible to obtain a interpretable set of structural connections, which are predictive for certain functional connections. An intrinsic, model-independent error measure of FC predictions was introduced to assure robust model selection. This process revealed interesting relationships between the underlying structural connections involved in shaping certain functional networks. In analogy to the randomized LASSO, a sparse CCA can be modified by using bootstrap with resampling to obtain a statistically interpretable mapping between SC and FC [74] and obtain connections which are consistently selected. Deligianni et al. [74] used this framework to relate different microstructural indices derived from DTI and neurite orientation dispersion and density imaging (NODDI) [222] to functional connectivity derived from a combination of EEG and fMRI. This allowed the authors to compare different types of microstructural indices with each other in the context of functional connectivity. In a subsequent study, Deligianni and Clayden [223] improved the CCA-based prediction by employing the transportation of FC matrices onto a Riemannian manifold. This ensured that the results of the linear prediction are restricted to be symmetric positive definite FC matrices, satisfying the criteria, when FC is estimated as a precision matrix, the inverse of the covariance matrix of time series. Similar to Deligianni et al., Reddi [47] also relied on sparse linear regression techniques to assess the SC-FC relationship. LASSO was used to predict the functional data of one ROI based on the data of all other ROIs. It was shown that the predictive performance was not affected when constraining the support to components with high structural connectivity, but strongly decreased when using components without any connections. When using regression to directly predict FC from SC, and also SC from FC, Reddi [47] showed that the structural connections could be better reconstructed from functional interactions, which might be due to the fact that tractography has difficulties to capture long-range interhemispherical connections [37].

In natural language processing, algorithms such as word2vec have been used to embed words into a vector space such that their vector representations capture syntactic and semantic word relationships [224, 225]. Rosenthal et al. [75] used a generalization of this method for network analysis to study the relationship between brain regions in the structural network. Instead of characterizing words in the context of sentences, algorithms such as node2vec can find representations of nodes in random walks within the network, which preserves the neighborhood relationships within the structural connectome [226]. These representations capture meaningful topological properties of the brain network and can be used for subsequent network analysis. In their study, Rosenthal et al. [75] used the embedding of structural network nodes to predict FC. At first, they incorporated a simple linear regression model for their SC-FC mapping, and in the next step, they showed that the mapping could be improved by employing a mulitlayer perceptron (MLP). Furthermore, they used this connectome embedding technique to predict the impact of lesions in the structural network on functional connectivity.

Contreras et al. [227] combined rs-fMRI with brain connectomics to characterize changes in whole-brain FC. Individual FC matrices were concatenated into a group FC matrix. The latter was decomposed with FastICA into ICs. Each of the resulting independent FC patterns was considered a response in a multilinear regression model including extraneous variables. Various ICs resulting from this connectivity independent component analysis (connICA), including RSN and DMN, were then further analyzed with respect to Alzheimer's disease. Recently, Amico and Goñi [73] proposed an extension to the connICA by decomposing hybridized structural and functional connectivity patterns. The independent, joint structural-functional patterns, extracted across a cohort of 100 datasets from the Human Connectome Project, represented two task-sensitive features, each encompassing connections within and between well-defined cortical areas. The integrated patterns can be considered connectivity fingerprints of a subject, deduced in a purely data-driven way.

Hence, despite considerable efforts to characterize dFC configurations deduced from rs-fMRI, the dynamics governing state transitions and their relationship to sFC still remains an open problem. Furthermore, the hypothesized latent brain states are yet to be related to the underlying SC. Surampudi et al. [76] recently proposed a graph-theoretic model that parameterized the low-dimensional manifold, which represents the temporal structure of functional connectivity patterns, by a set of local density distributions and learned the parameters from data via a temporal multiple-kernel learning (tMKL) strategy. The latter directly links dynamics to the underlying structure via a state-transition Markov model (see Figure 8). Finally, the model predicts the grand average FC across a group of subjects but retains sensitivity towards subject-specific anatomy. The model was tested using the rs-fMRI data of 46 healthy participants, and its generalizability was proven through a test on a cohort of 100 subjects from the Human Connectome Project. The authors claimed that their tMKL model performs considerably better than a whole-brain dynamic mean-field (DMF) model [84], a single diffusion kernel (SDK) model [48], or a multiple-kernel learning (MKL) model [228].

## 6. Conclusion

### 6.1. Current Methods

Bridging the gap between dynamic brain functions and their relatively static structural backbone is one of the keys for understanding the underlying mechanisms which drive information processing in the human brain and are physically confined by the anatomical substrate of neurons, nerve fibers, and synapses. Accordingly, recent research has focused mainly on the resting-state paradigm, which excludes external stimuli to elicit functional dynamics. Such a focus might bear the risk of introducing a strong bias towards this paradigm. It is apparent that even at rest, the structure-function relation is highly complex and will remain a subject of intense research in the near future, but for the long term, it could also be of interest to study the impact of stimuli or task-based paradigms on this relationship [16].

Currently, methods from graph theory are used to map functional onto structural connectivity, highlighting the importance of indirect structural connections for modeling the spread of neural activity [53, 55, 59]. Such approaches showed that it is possible to explain functional connectivity patterns, to a relatively large extent, alone from its structural backbone, given proper constraints [54], but computing only temporal correlations of activities in different brain regions was shown to result in a certain loss of information. For such graph theory-inspired approaches, it might therefore be necessary in the future to rather rely on dynamic FC instead of static FC only [76] to successfully characterize the relationship between SC and FC.

So far, models related to computational connectomics provided insights into different aspects of brain dynamics, emerging on various time scales [16, 83]. Computational approaches helped to further interpret these complex brain dynamics by employing concepts found in statistical physics [2, 60, 111, 229]. They showed that the resting brain can be seen as a system operating at a point of maximum metastability [2], where resting-state dynamics are driven by fluctuations around this critical point. In terms of interpretability, these models can be very informative because they are based on meaningful and well-understood physical processes. On the contrary, it is still challenging to design models, which meet the requirements of explaining the complex empirically observed activation patterns in the brain, thus bridging the gap between spiking neurons and whole-brain dynamics [78]. Also, their explanatory value is often confined to a priori-defined models and assumptions. Machine learning methods could add valuable support for research in this field by providing novel insights from a data-driven perspective.

Another largely unexplored aspect of structure-function relationships concerns the impact of different acquisition and preprocessing schemes. As there are, nowadays, various sequences established to acquire fMRI and DTI data, as well as elaborate pipelines for further processing, the choice of data acquisition and processing seems to have a considerable impact on the strength on the SC and FC relationship [56, 61, 230]. Therefore, it might be necessary to also question the concepts, which even define structural and functional connectivity, and which concept is most appropriate for the above-discussed questions. In many preprocessing pipelines, the variance of SC across subjects is relatively weak, as shown by Zimmermann et al. [61]. This observation immediately raises the question of how much information the subject-specific SC contains about its related FC when extracted with current methods. Several efforts have already been made to infer such a mapping on the individual subject level [53, 55, 228]. However, for such applications, several studies have indicated that a more fine-grained parcellation of the cortex might be necessary for the emergence of subject-specific features [4, 47, 61]. Also, alternatives in structural imaging techniques such as neurite orientation dispersion and density imaging (NODDI) [222] or diffusion kurtosis imaging (DKI) [231] could provide additional insights into the white matter architecture. Finally, as fMRI is limited in the temporal resolution, usually around 1 to 2 seconds, it is clear that we do not get the complete information about brain dynamics from this imaging modality alone. In future, additional information from faster modalities such as EEG and MEG will be crucial in order to overcome these limitations of MRI and help to further close the gap between the brain structure and its function [16, 57]. For example, simultaneous EEG-fMRI techniques gain already more and more attention in multimodal studies and clinical assessments [232, 233]. Such data fusion approaches could also contribute valuable insights into the structure-function relation because they would allow us to observe the propagation of neural signals onto the anatomical substrate at considerably higher temporal resolution than in fMRI alone. Therefore, incorporating multimodal imaging techniques will likely be a key aspect in our efforts to obtain a more comprehensive picture of neural connectivity in the human brain.

### 6.2. Potential Future Directions of ML Applications

Machine learning has already proven to be useful for identifying functionally independent networks [117], but it also has emerged, more recently, as a tool for learning about the structure-function relationship. While computational simulations tell us much about the mechanisms of brain dynamics, every model relies on specific assumptions concerning the physical nature of these dynamics. On the contrary, data-driven methods from machine learning might not be able to replicate natural processes in the brain, but nevertheless can adapt to complex statistical data structures intrinsically. By studying features, learned by data-driven models, interesting structure-function relations can be revealed, such as which structural connections are involved in shaping the functional connectivity strength between two brain areas [74]. Also, hybrid models showed considerable potential in revealing new statistical features buried in functional imaging data [129]. By making relatively general assumptions, such as statistical independence in ICA or sparseness like in the LASSO, a lot of freedom is given to explore the inherent relationships in empirical data. This approach can be used to generate a novel hypothesis, which might have been largely unexplored while solely relying on predefined models. Therefore, machine learning approaches are not competing with computational or graph theoretical inspired models, but rather they supplement current methods.

Most of the above-discussed machine learning methods rely mainly on classical data-driven approaches, but following a recent trend, also, deep learning (Deep learning refers to neural network models, which extract features from input stimulus patterns at various levels of spatial and/or temporal resolution in subsequently deeper layers of the network. In general, simple features are extracted at high resolution but small scale, while complex features are extracted at low resolution and larger scales.) [234] has gained increasingly more attention in the neuroscience community [235, 236]. If sufficient data are available, such models are capable of finding highly nonlinear patterns in various data types, without much prior knowledge being required about the structures underlying the data. On the downside, the high level of abstraction of the data representation in various layers of such deep neural networks renders it rather hard to understand which patterns in the data are relevant for generating a specific hypothesis. Therefore, the explanatory value of such models is quite controversial, and they will not provide the same detailed insights into neural processes such as computational models based on mathematically well-defined mechanisms and concepts. However, their exploratory capabilities make it already an attractive tool for numerous scientific questions [235] and could give novel insight into the complex relationship between the brain structure and function. Even more recently, some efforts have been made in order to make these machine learning models more transparent. The SHAP (SHapleyAdditive exPlanations) framework, for example, presents a unified method for quantifying feature importance in order to bridge the gap between model accuracy and interpretability [237, 238].

In summary, decomposing, in a purely data-driven manner, complex activity patterns into underlying features, relevant on various spatial or temporal scales, will certainly help to formulate proper assumptions and constraints, used with computational models to further understand of the underlying physical mechanisms leading to the observed complex nonstationary activity patterns.

## Figures and Tables

**Table 1:** Temporal and spatial scales of several neuroimaging techniques.

Method	Resolution	
	Temporal	Spatial
MEGs	1 ms	5 mm
EEG	1 ms	10–15 mm
fMRI	1 s	1–3 mm
PET	45 s	4 mm

## Appendix

### A. Graph Laplacian

#### A.1. Adjacency Matrix

An adjacency matrix **A** is a square matrix, whose elements indicate whether pairs of vertices (*v*(*n*), *v*(*n*′)) are adjacent or not in a graph, often denoted as *n* ~ *n*′. A simple graph is represented by an adjacency matrix, whose entries are *a*_*nn*′_={0,1} and which has all zeros along its diagonal. A weighted adjacency matrix **A**_*w*_, instead, contains weights *a*_*nn*′_=*w*_*nn*′_ for each edge excluding self-couplings. In an undirected graph, the adjacency matrix is a real symmetric semipositive matrix, i.e., *a*_*nn*′_=*a*_*n*′*n*_ ≥ 0. The relationship between a graph and the eigendecomposition of its adjacency matrix is studied in spectral graph theory.

#### A.2. Degree Matrix

The diagonal degree matrix **D**=diag(**A**_*w*_1) contains information about the number of edges emanating from any vertex, i.e., given a graph *𝒢*=(*𝒱*, *ℰ*) with |*V*|=*N*, **D** ∈ *ℝ*^*N*×*N*^ and

(A.1) $$\begin{array}{c} d_{nn^{\prime }}=\left\{\begin{array}{cc} d_{nn} & \text{if }n=n^{\prime },\\ 0, & \text{otherwise,} \end{array}\right.\\ d_{nn}=\sum_{n^{\prime }}w_{nn^{\prime }}, \end{array}$$

where *d*_*nn*_=deg(*v*_*n*_) and *w*_*nn*′_ denote elements of the weighted adjacency matrix.

#### A.3. Graph Laplacian

The Laplacian matrix is a matrix representation of a graph. It is also called admittance matrix, Kirchhoff matrix, and discrete Laplacian. Given a weighted graph with *N* vertices or nodes, its Laplacian matrix is defined as

(A.2) $$\begin{array}{c} L=D-A,\\ L_{nn^{\prime }}=\left\{\begin{array}{cc} d_{nn}=\underset{n^{\prime }}{\sum }w_{nn^{\prime }} & \text{if }n=n^{\prime },\\ -w_{nn^{\prime }} & \text{if }n\neq n^{\prime }\text{ and }v_{n}\text{ is adjacent to }v_{n^{\prime }},\\ 0, & \text{otherwise.} \end{array}\right. \end{array}$$

In case of an undirected graph, Laplacian is a symmetric semipositive matrix with its associated eigendecomposition:

(A.3) $$\begin{array}{c} U^{T}LU=\Lambda ⟺LU=U\Lambda ⟺L=U\Lambda U^{T}, \end{array}$$

where **U**=(**u**_1_,…., **u**_*N*_) denotes the eigenvector matrix and Λ=diag(*λ*_1_,…, *λ*_*N*_) is the related eigenvalue matrix. In case of directed graphs, either the in-degree or out-degree might be used.

The symmetric normalized Laplacian matrix is defined as

(A.4) $$\begin{array}{c} L_{n}=D^{-1/2}LD^{-1/2}=I-D^{-1/2}AD^{-1/2},\\ {\left(L_{n}\right)}_{nn^{\prime }}=\left\{\begin{array}{cc} 1-\frac{w_{nn}}{d_{nn}} & \text{if }n=n^{\prime }\text{ and }d_{nn}=\underset{n^{\prime }}{\sum }w_{nn^{\prime }}\neq 0,\\ -\frac{w_{nn^{\prime }}}{\sqrt{d_{nn}d_{n^{\prime }n^{\prime }}}} & \text{if }n\neq n^{\prime }\text{ and }v_{n}\text{ is adjacent to }v_{n^{\prime }},\\ \text{0,} & \text{otherwise.} \end{array}\right. \end{array}$$

The eigendecomposition of the graph Laplacian **L** and its normalized variant **L**_*n*_ is related by

(A.5) $$\begin{array}{c} L_{n}=D^{-1/2}LD^{-1/2}=D^{-1/2}U\Lambda U^{T}D^{-1/2}=\hat{U}\Lambda {\hat{U}}^{T}, \end{array}$$

(A.6) $$\begin{array}{c} \hat{U}=D^{-1/2}U, \end{array}$$

(A.7) $$\begin{array}{c} \Lambda =\text{diag}\left({\left\{\lambda_{l}\right\}}_{l=1}^{N}\right), \end{array}$$

where $\hat{U}=\left({\hat{u}}_{1},\ldots ,{\hat{u}}_{N}\right)$ denotes the complete set of orthogonal eigenvectors. Note that the graph Laplacian and its normalized variant possess the same eigenvalues.

#### A.4. Transition Matrix and Random Walk Graph Laplacian

A random walk on a graph can be modeled as a Markov process employing the transition matrix **L**_*t*_, which describes transitions between adjacent and connected nodes.

(A.8) $$\begin{array}{c} L_{t}=D^{-1}A_{w}=\left(\begin{array}{ccc} {\hat{w}}_{11} & \cdots & {\hat{w}}_{1N}\\ \vdots & {\hat{w}}_{nn} & \vdots \\ {\hat{w}}_{N1} & \cdots & {\hat{w}}_{NN} \end{array}\right),\\ {\hat{w}}_{nn}=\frac{w_{nn}}{\sum_{n^{\prime }}w_{nn^{\prime }}}. \end{array}$$

Note that *w*_*nn*′_ ≠ 0 only if vertices *v*_*n*_ and *v*_*n*′_ are adjacent and connected. The related random walk graph Laplacian is then defined as

(A.9) $$\begin{array}{c} L_{rw}=D^{-1}L=D^{-1}\left(D-A_{w}\right)=I-D^{-1}A_{w}=I-L_{t}=\left(\begin{array}{ccc} 1-\frac{w_{11}}{d_{11}} & \cdots & -\frac{w_{1N}}{d_{11}}\\ \vdots & 1-\frac{w_{nn}}{d_{nn}} & \vdots \\ -\frac{w_{N1}}{d_{NN}} & \cdots & 1-\frac{w_{NN}}{d_{NN}} \end{array}\right). \end{array}$$

Note the similarity of the random walk Laplacian to the normalized graph Laplacian:

(A.10) $$\begin{array}{c} L_{rw}=D^{-1/2}L_{n}D^{1/2}. \end{array}$$

Hence, we have that **L**_*rw*_ ~ **L**_*n*_ because **D**^1/2^ is an invertible matrix and thus induces a similarity transformation. Consequently, the random walk graph Laplacian has the following eigendecomposition:

(A.11) $$\begin{array}{c} L_{rw}=D^{-1/2}L_{n}D^{1/2}=D^{-1/2}\hat{U}\Lambda {\hat{U}}^{T}D^{1/2}=D^{-1}U\Lambda U^{T}. \end{array}$$

### B. Matrix Decomposition Techniques

#### B.1. Singular Value Decomposition and Principal Component Decomposition

Given a centered data matrix **X** ∈ *ℝ*^*N*×*M*^, *N* ≥ *M*, with zero mean, and let *N* represent the spatial dimension and *M* the temporal dimension. Its singular value decomposition (SVD) then reads

(B.1) $$\begin{array}{c} X=U\Sigma V^{T}, \end{array}$$

whereby **U** represents the orthogonal eigenvector matrix of the related outer product correlation matrix **C**=**X****X**^*T*^.

(B.2) $$\begin{array}{c} C=U\Lambda_{C}U^{T}=U\Sigma_{C}\cdot {\left[U\Sigma_{C}\right]}^{T}. \end{array}$$

This decomposition is called principal component analysis (PCA). Furthermore, Λ_*C*_=Σ^2^ denotes the matrix of related eigenvalues *λ*_*i*_=*σ*^2^ with *σ*_*i*_ being the corresponding singular values. Finally, **V** represents the orthogonal eigenvector matrix of the related inner product kernel matrix **K**=**X**^*T*^**X**.

(B.3) $$\begin{array}{c} K=V\Lambda_{K}V^{T}=V\Sigma_{K}\cdot {\left[V\Sigma_{K}\right]}^{T}. \end{array}$$

Note that the first *M* eigenvalues of both Λ_*K*_ and Λ_*C*_ are identical.

#### B.2. Independent Component Decomposition

Given a centered data matrix **X** ∈ *ℝ*^*N*×*M*^, *N* ≥ *M*, with zero mean, its independent component decomposition reads

(B.4) $$\begin{array}{c} X\approx AS, \end{array}$$

whereby rows $\tilde{s_{l}}$ of component matrix **S** shall be as statistically independent as possible. Note that a perfect factorization might not be possible always. With fMRI, ICA comes in two flavors, spatial (sICA) and temporal (tICA) independent component analysis. With sICA,  ^**s**^**S** represents independent spatial maps, while with tICA,  ^**t**^**S** represents independent voxel time series. A spatiotemporal blind signal decomposition tries to decompose the data matrix into a product of two independent factor matrices [139].

(B.5) $$\begin{array}{c} X\approx^{t}S^{s}S. \end{array}$$

#### B.3. Nonnegative Matrix Factorization

Given a nonnegative data matrix **X** ∈ *ℝ*^*N*×*M*^, *N* ≥ *M*, nonnegative matrix factorization amounts to finding two component matrices **W**, **H** with strictly nonnegative entries only. We then have

(B.6) $$\begin{array}{c} X\approx WH. \end{array}$$

Here, the rows of the component matrix **H** ∈ *ℝ*^*L*×*M*^ represent characteristic local time courses, and the corresponding rows of the component matrix **W** reflect the weights with which these component time courses contribute to each observed time course in **X**.

#### B.4. Empirical Mode Decomposition

Given a centered data matrix **X** ∈ *ℝ*^*N*×*M*^, *N* ≥ *M*, with zero mean, its decomposition into intrinsic modes yields

(B.7) $$\begin{array}{c} X=\sum_{r=1}^{R}S^{\left(r\right)}, \end{array}$$

whereby **S**^(*r*)^ represents intrinsic component matrices with characteristic spatial frequencies reflecting intrinsic textures contained in the component matrices. Each intrinsic mode represents pure 2D spatial oscillations occurring on characteristic time scales except the last mode, which represents a nonoscillation trend.

### C. Hidden Markov Models

The idea of a hidden Markov model is to make inferences on a system, characterized by a set of hidden states *H*=[*h*_1_, *h*_2_,…, *h*_*N*_], by observing a sequence of emitted states *X*=[*x*_1_, *x*_2_,…, *x*_*T*_], with *X* depending on *H*. These models are described by the following two properties.

Hidden states *H* follow a Markov process:

(C.1) $$\begin{array}{c} P\left(h_{i}|h_{1},\ldots ,h_{i-1}\right)=P\left(h_{i}|h_{i-1}\right), \end{array}$$

and the emission probability of *x*_*i*_ only depends on the systems' current state *h*_*i*_:

(C.2) $$\begin{array}{c} P\left(x_{i}|h_{1},\ldots ,h_{i}\ldots ,h_{N},x_{1},\ldots ,x_{i},\ldots ,x_{T}\right)=P\left(x_{i}|h_{i}\right). \end{array}$$
